# Supplementary material for: Polerovirus N-terminal readthrough domain structures reveal molecular strategies for mitigating virus transmission by aphids
Source: Nat Commun. 2022 Oct 26;13:6368. doi: 10.1038/s41467-022-33979-2 (PMC9606263; doi:10.1038/s41467-022-33979-2)
Supplement: Supplementary file 1 — Supplementary Information [file 41467_2022_33979_MOESM1_ESM.pdf]

Supplementary information for:

Polerovirus N-terminal readthrough domain structures reveal molecular strategies for mitigating virus transmission by aphids

Carl J. Schiltz[1,2‡], Jennifer R. Wilson[3,4‡], Christopher J. Hosford[1,5], Myfanwy C. Adams[1], Stephanie E. Preising[3], Stacy L. DeBlasio[3,6], Hannah J. MacLeod[6,7], Joyce Van Eck[8,9], Michelle L. Heck[3,6,9*] and Joshua S. Chappie[1,*]

[1] Department of Molecular Medicine, Cornell University, Ithaca, NY, 14853, USA

[2] Present address: Department of Biological Sciences, Vanderbilt University, Nashville, TN, 37232, USA

[3] Section of Plant Pathology and Plant-Microbe Biology, School of Integrative Plant Sciences, Cornell University, Ithaca, NY, 14853, USA

[4] Present address: USDA-Agricultural Research Service, Corn, Soybean & Wheat Quality Research Unit, Wooster, OH, 44691, USA

[5] Present address: LifeMine Therapeutics, Cambridge, MA 02140, USA

[6] USDA-Agricultural Research Service, Emerging Pest and Pathogen Research Unit, Ithaca, NY, 14853, USA

[7] Present address: Accelevir Diagnostics, Baltimore, MD 21202, USA

[8] Section of Plant Breeding and Genetics, School of Integrative Plant Sciences, Cornell University, Ithaca, NY 14853, USA

[9] Boyce Thompson Institute for Plant Research, Ithaca, NY, 14853, USA

* To whom correspondence should be addressed. Email: chappie@cornell.edu, mlc68@cornell.edu

‡ Authors contributed equally

Supplementary Figures

Fig. S1. Domain architecture and purification of ^{N}RTD constructs. Related to Fig. 1.

Fig. S2. Subunit organization and symmetry of TBSV and PLRV viral capsids. Related to Figs. 2 and 3.

Fig. S3. Structure and topology of the PLRV ^{N}RTD. Related to Fig. 1.

Fig. S4. Cap domain β-barrel adopts a conserved fold with unique topology. Related to Figs. 1 and 2.

Fig. S5. Sequence alignment of the ^{N}RTD region across poleroviruses, enamoviruses, and luteoviruses.

Fig. S6. ^{N}RTD structures resolve clear density for the C peptide. Related to Figs. 1 and 2.

Fig. S7. Architecture of the PLRV ^{N}RTD dimer. Related to Fig. 2.

Fig. S8. Dimensions of TuYV NRTD dimer. Related to Figs. 2 and 3.

Fig. S9. Structural mapping of polerovirus, enamovirus, and luteovirus sequence conservation. Related to Figs.1 and 2.

Fig. S10. Location and structural stability of PLRV ^{N}RTD mutants from previous literature. Related to Fig. 2.

Fig. S11. Experimental design for artificial diet feeding experiments. Related to Figs. 4 and 5.

Fig. S12. Transient *in planta* delivery of the PLRV ^{N}RTD to aphids. Related to Fig. 4.

Fig. S13. Transgenic potato delivery of the ^{N}RTD. Related to Fig. 4.

Fig. S14. Metanalysis and forest plot of all WT PLRV ^{N}RTD trials. Related to Fig. 4.

Fig. S15. Location of PLRV cap domain mutants. Related to Fig. 5.

Supplementary Tables

Table S1. X-ray data collection and refinement statistics.

Table S2. Summary of PLRV and TuYV ^{N}RTD reverse genetic studies from the literature.

Table S3. PLRV transmission by *M. persicae* aphids after artificial diet delivery of PLRV ^{N}RTD and H321A.

Table S4. Logistic regression analysis of PLRV transmission by *M. persicae* aphids after artificial diet delivery of PLRV ^{N}RTD and H321A.

Table S5. PLRV transmission by *M. persicae* aphids after transient *in planta* delivery of PLRV ^{N}RTD.

Table S6. Logistic regression analysis of PLRV transmission by *M. persicae* aphids after transient *in planta* delivery of PLRV ^{N}RTD.

Table S7. PLRV transmission by *M. persicae* aphids after transgenic potato delivery of PLRV ^{N}RTD.

Table S8. Logistic regression analysis of PLRV transmission by *M. persicae* aphids after transgenic potato delivery of PLRV ^{N}RTD.

Table S9. Mortality of *M. persicae* aphids after artificial diet delivery of PLRV ^{N}RTD mutants.

Table S10. Quasibinomial regression analysis of *M. persicae* mortality after artificial diet delivery of PLRV ^{N}RTD mutants.

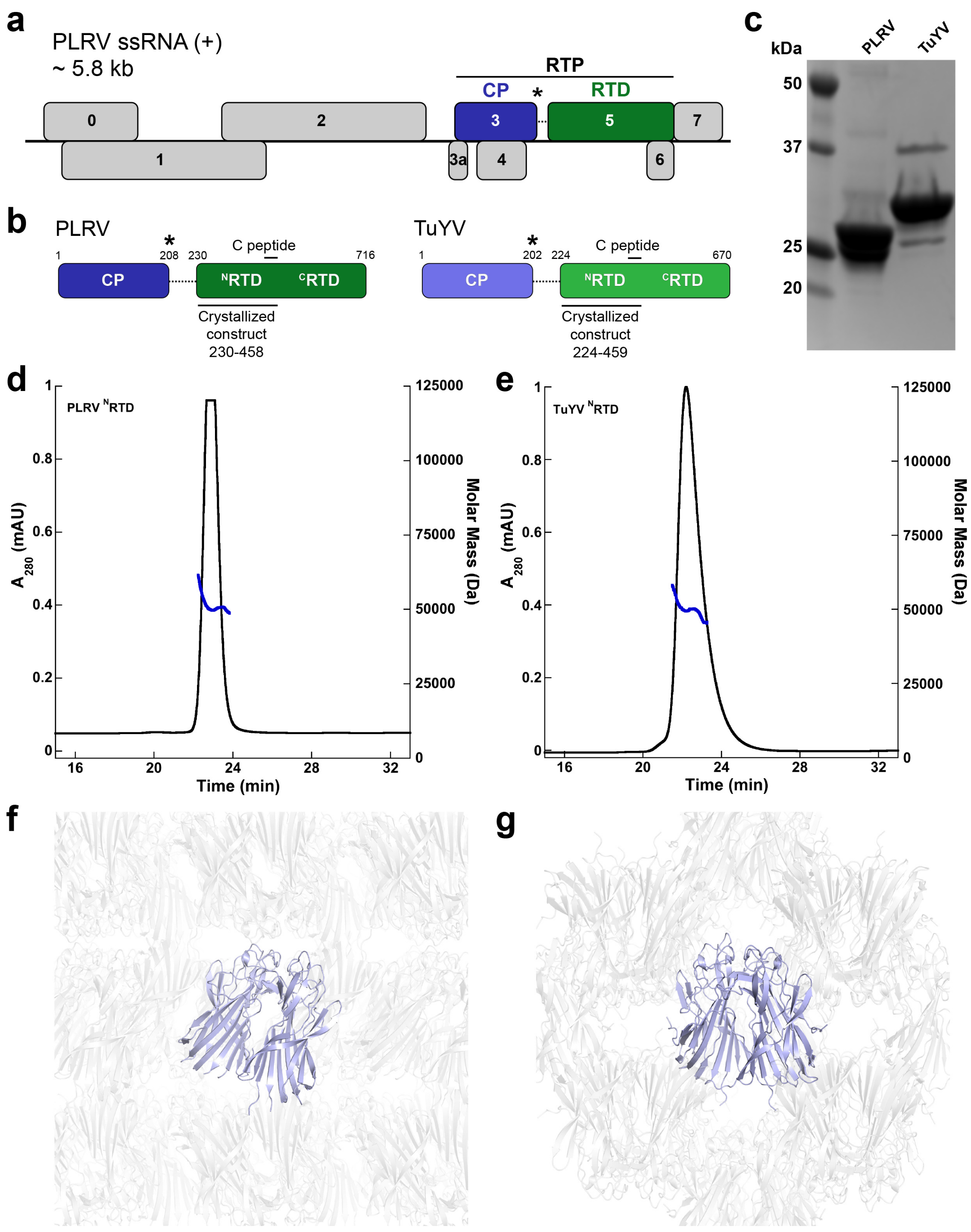

Fig. S1. Domain architecture and purification of polerovirus ^{N}RTD constructs. a, PLRV genome arrangement. ORFs 3 (coat protein, CP) and 5 (readthrough domain, RTD) together encode the viral readthrough protein (RTP). b, Domain arrangement of PLRV and TuYV readthrough proteins. CP and RTD segments are labeled and connected by a variable linker

region (dashed line, see also Fig. S5). Domain boundaries of the CP and RTD are derived from structural studies[1,2]. The relative location of the crystallized ^{N}RTD construct and C peptide in each RTP are marked. Asterisk denotes position of the leaky stop codon (immediately following 208 in PLRV and 202 in TuYV) that when bypassed yields the full RTP. c, SDS-PAGE gel of purified ^{N}RTDs. First lane contains molecular weight markers (kDa). Arrowhead denotes position of bands. Gel representative of 10 independently expressed and purified batches of protein. d-e, SEC-MALS analysis of purified PLRV (d) and TuYV (e) ^{N}RTD constructs. Black line denotes UV trace and blue line denotes measured mass across each peak. Calculated molecular weights of PLRV and TuYV ^{N}RTD monomers are 26.4 kDa and 27.1 kDa respectively. f-g, Crystal packing of PLRV (f) and TuYV (g) ^{N}RTD constructs. Dimers are colored light blue.

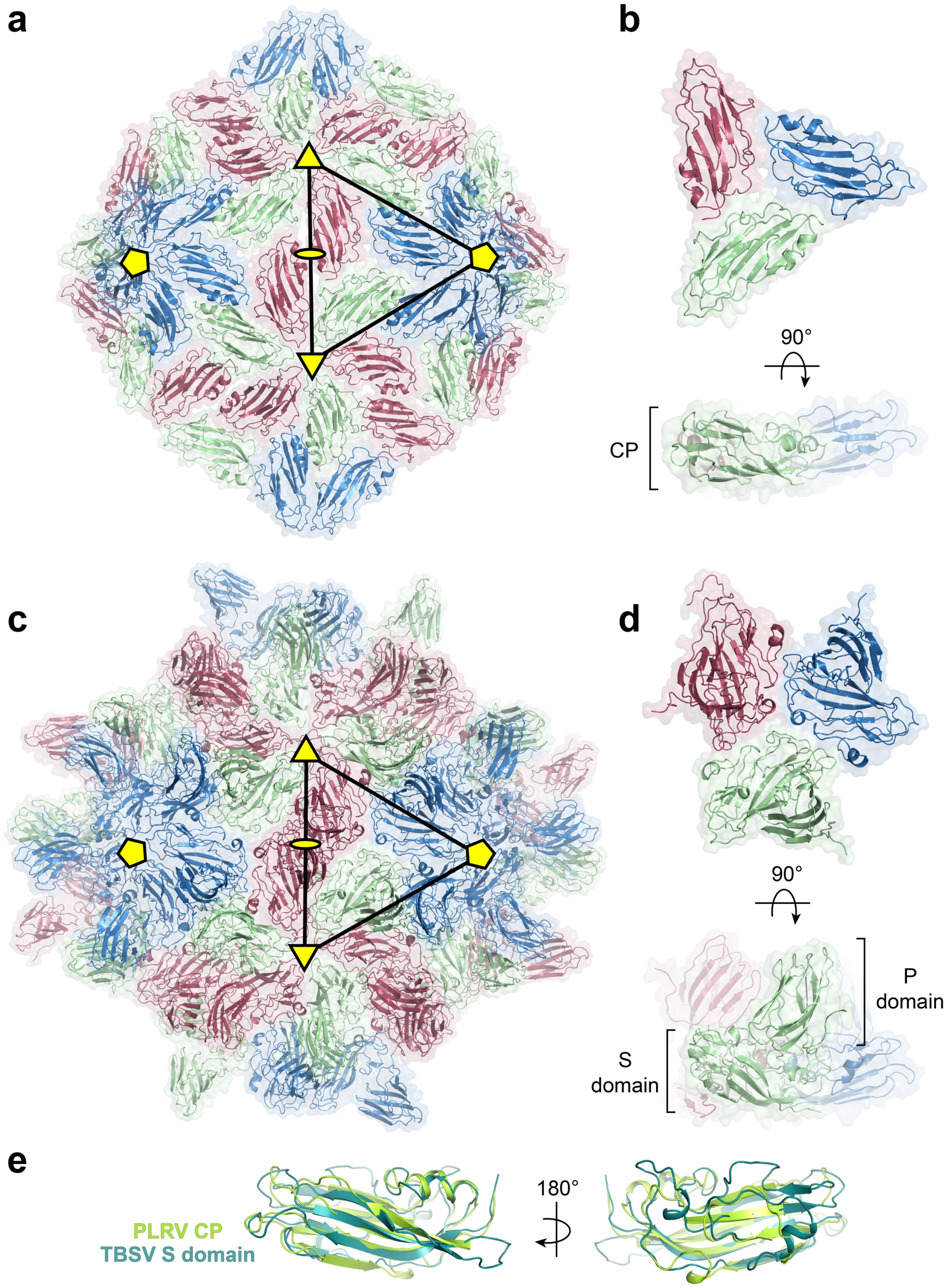

Fig. S2. Subunit organization and symmetry of PLRV and TBSV viral capsids. a, T=3 icosahedral symmetry of PLRV capsid (PDB: 6SCO [http://doi.org/10.2210/pdb6SCO/pdb]). Individual subunits that constitute the icosahedral asymmetric unit (black triangles) are colored raspberry, light green, and sky blue respectively. Two-, three-, and five-fold symmetry axes are marked with a yellow ellipse, yellow triangles, and yellow pentagons, respectively. b, Top and

side views of the isolated PLRV asymmetric unit. The PLRV coat protein is labeled. c, T=3 icosahedral symmetry of TBSV capsid (PDB: 2TBV [http:doi.org/10.2210/pdb2TBV/pdb]). Individual subunits that constitute the icosahedral asymmetric unit are colored as in a. d, Top and side views of the TBSV asymmetric unit. S and P domains are labeled. e, Superposition of PLRV CP (lime) and TBSV S domain (teal) monomers.

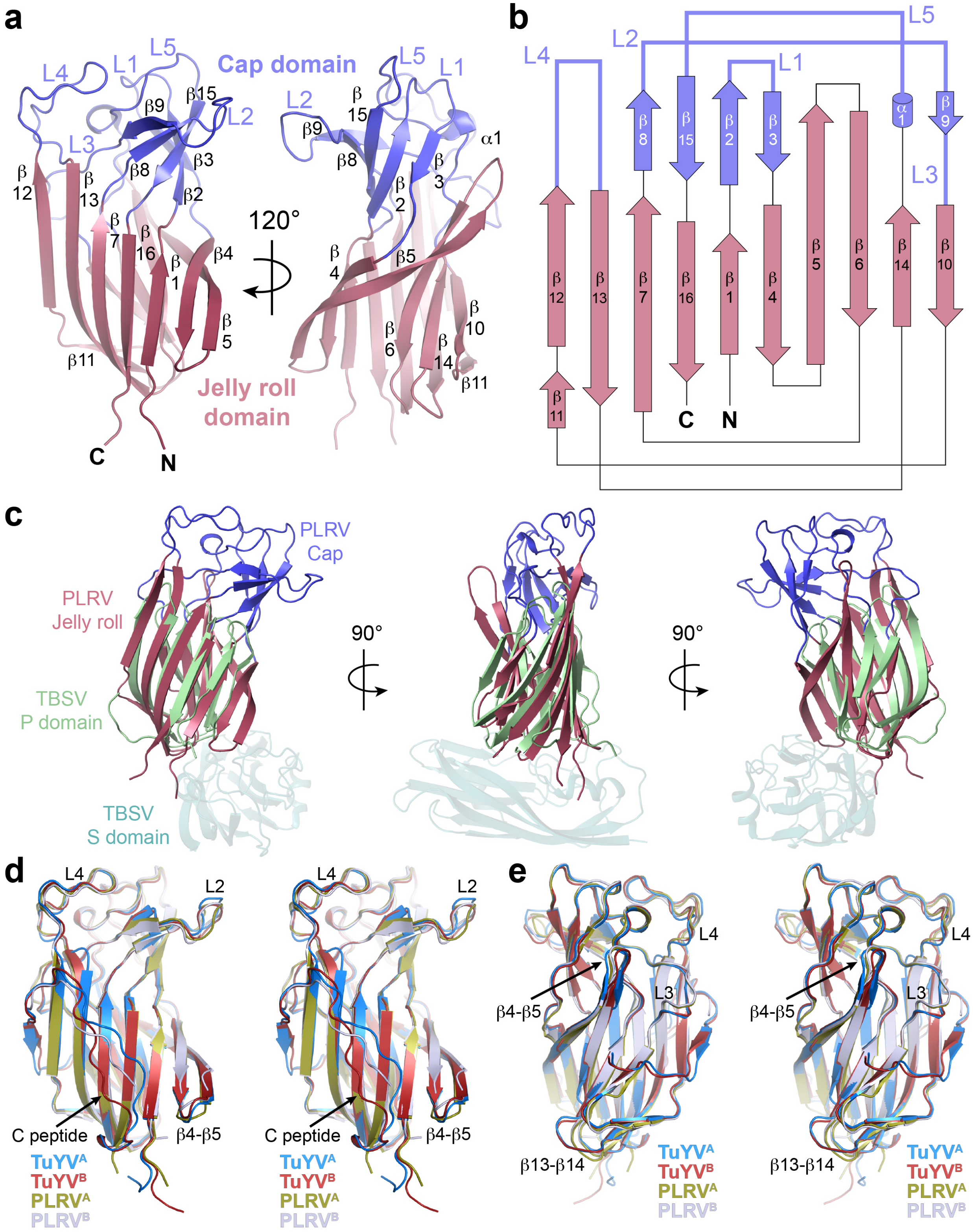
a
Cap domain
Jelly roll domain
L1
L2
L3
L4
L5
120°
C
N
b
α1
c
PLRV Cap
PLRV Jelly roll
TBSV P domain
TBSV S domain
90°
90°
d
C peptide
β4-β5
TuYVA
TuYVB
PLRVA
PLRVB
e
β4-β5
β13-β14
TuYVA
TuYVB
PLRVA
PLRVB

Fig. S3. Structure and topology of the PLRV ^{N}RTD. a, Structure (a) and topology (b) of PLRV ^{N}RTD with jelly roll domain (red), cap domain (blue) labeled. Cap domain loops are labeled L1-L5. See Fig. S5 for correspondence between the secondary structure elements and the PLRV sequence. c, Superposition of PLRV ^{N}RTD and tomato bushy stunt virus (TBSV) coat protein (PDB: 2TBV [http:doi.org/10.2210/pdb2TBV/pdb]; sequence identity: 4% (across the P domain); DALI[3] Z score: 8.3; RMSD: 2.5 Å; P domain, light green, S domain, teal). d-e, Superposition of TuYV (marine and red) and PLRV (olive and bluewhite) ^{N}RTD monomers shown in stereo in two orientations. Segments exhibiting conformational differences are labeled in black.

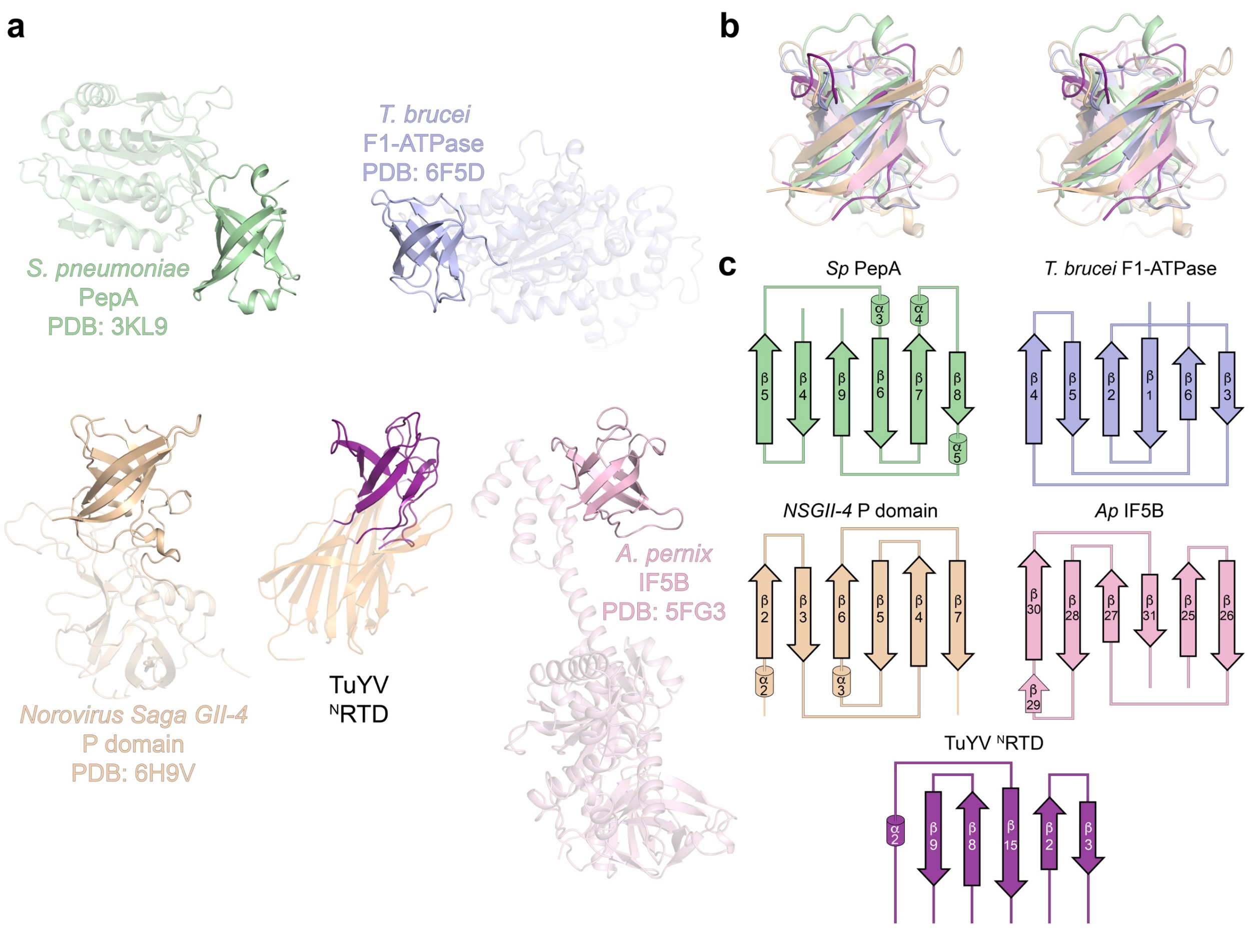

Fig. S4. Cap domain β-barrel adopts a conserved fold with unique topology. a, TuYV ^{N}RTD cap domain (purple) shares a conserved β-barrel fold that is present in a wide array of proteins. Structural representatives are shown with associated PDB codes [http://doi.org/10.2210/pdb3KL9/pdb; http://doi.org/10.2210/pdb6F5D/pdb; http://doi.org/10.2210/pdb6H9V/pdb; http://doi.org/10.2210/pdb5FG3/pdb]. b, Superposition of β-barrel domains shown in stereo with individual models colored as in (a). c, Topology diagrams of conserved β-barrel domains depicted in (a) and (b).

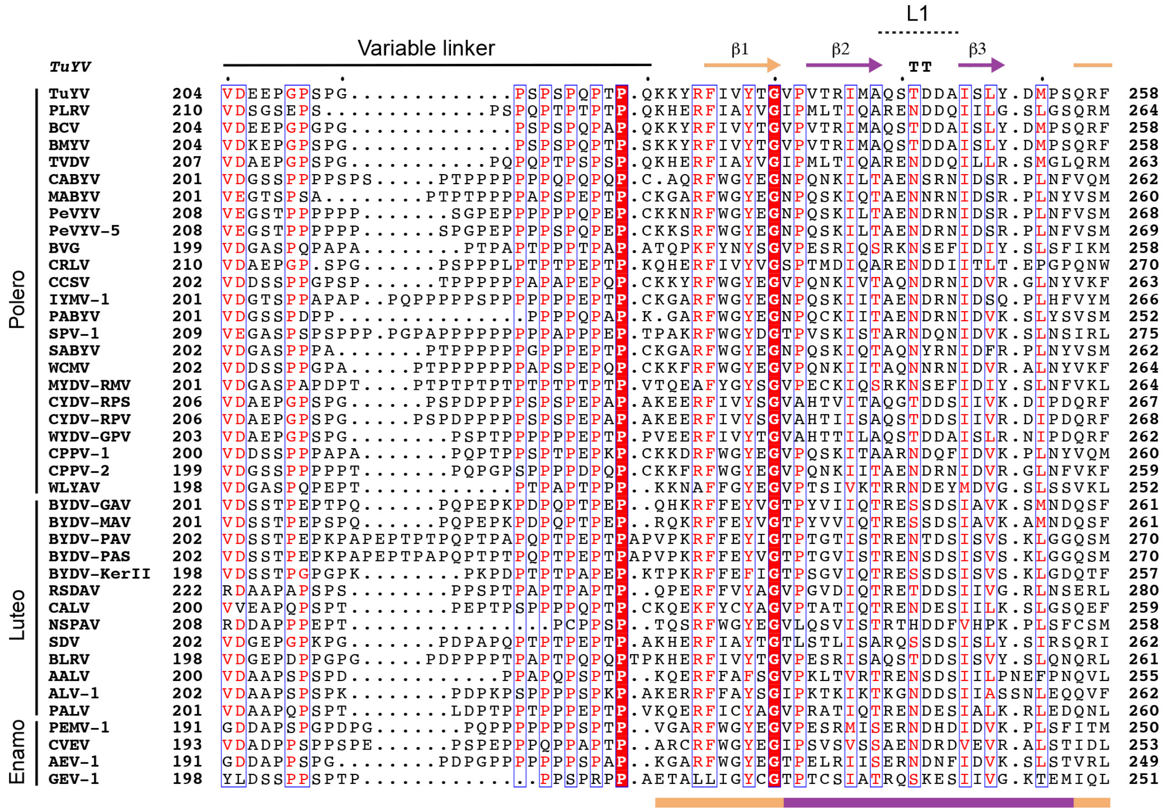
Variable linker
L1
β1
β2
β3
TT
TuYV
Polero
Luteo
Enamo
TuYV
PLRV
BCV
BMYV
TVDV
CABYV
MABYV
PeVYV
PeVYV-5
BVG
CRLV
CCSV
IYMV-1
PABYV
SPV-1
SABYV
WCMV
MYDV-RMV
CYDV-RPS
CYDV-RPV
WYDV-GPV
CPPV-1
CPPV-2
WLYAV
BYDV-GAV
BYDV-MAV
BYDV-PAV
BYDV-PAS
BYDV-KerII
RSDAV
CALV
NSPAV
SDV
BLRV
AALV
ALV-1
PALV
PEMV-1
CVEV
AEV-1
GEV-1

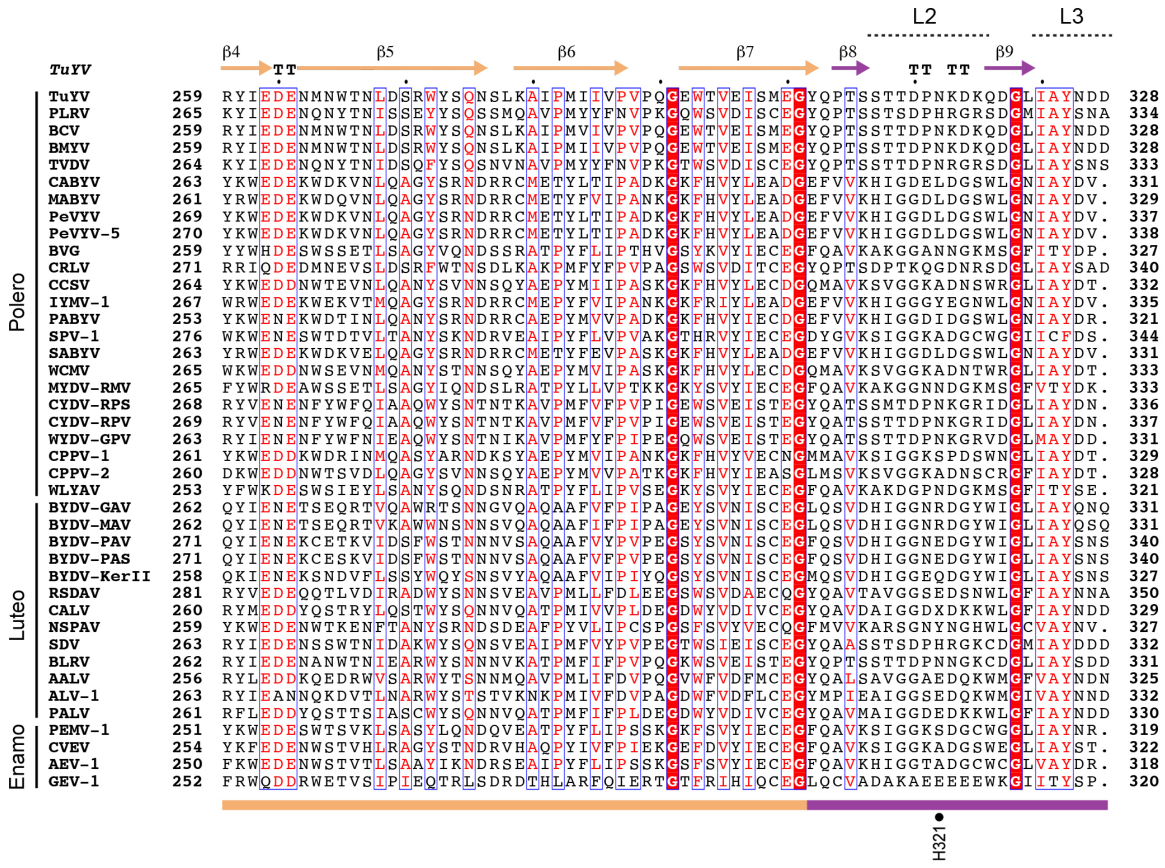
L2
L3
β4
β5
β6
β7
β8
β9
TT
TT TT
TuYV
Polero
Luteo
Enamo
H321

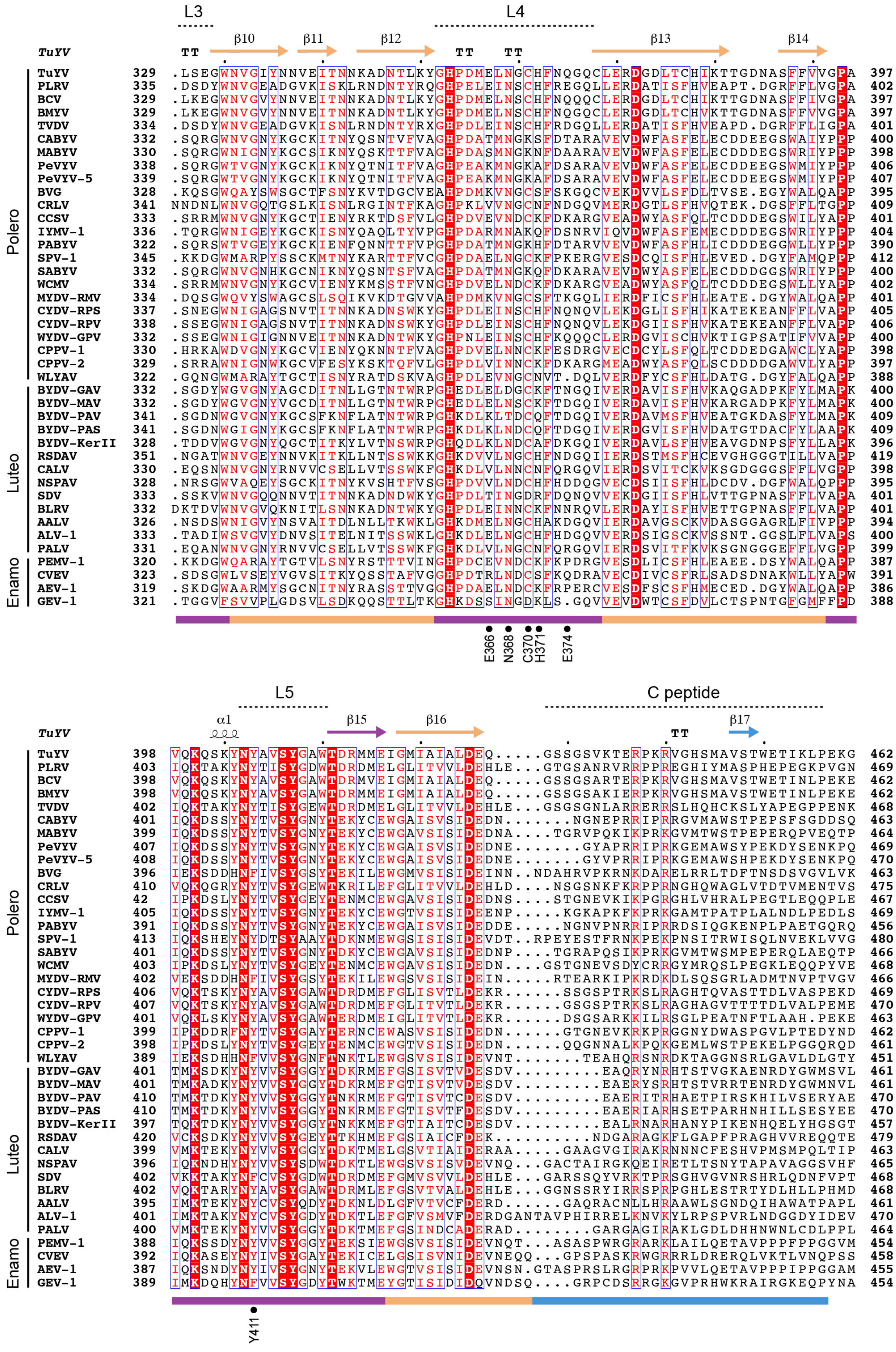
L3
L4
β10
β11
β12
β13
β14
TuYV
Polero
Luteo
Enamo
E366
N368
C370
H371
E374
L5
C peptide
α1
β15
β16
β17
Y411

Fig. S5. Sequence alignment of polerovirus, enamovirus, and luteovirus ^{N}RTDs. Sequence alignment of corresponding ^{N}RTD regions with the secondary structure of the TuYV ^{N}RTD mapped above. Sequences are organized by genera to distinguish between poleroviruses (Polero), luteoviruses (Luteo), and enamoviruses (Enamo). Orange-, purple-, and marine-colored bars beneath the alignment denote segments within the jelly roll domain, cap domain, and C peptide respectively (see Fig. 1). Cap domain loops (L1-5) and C peptide are labeled above along with the variable linker that connects the CP to the ^{N}RTD (see Fig. S1b). Black circles below the alignment designate positions of PLRV point mutations tested in the aphid transmission and mortality assays (see Figs. 4 and 5 and Tables S4, S9, and S10). Sequence shading indicates conservation: white text on red background, 100% conserved; boxed red text on white background, 70% conserved. Sequence numbering for each virus considers readthrough codon as an additional residue. Abbreviations are as follows with accompanying KEGG IDs and calculated percent sequence identity/similarity[4] for each virus compared to TuYV: TuYV, turnip yellows virus (vg:940480 [https://www.genome.jp/entry/vg:940480]); PLRV, potato leafroll virus (vg:1493889 [https://www.genome.jp/entry/vg:1493889]; 47.91%/63.50%); BCV, beet chlorosis virus (vg:921081 [https://www.genome.jp/entry/vg:921081]; 94.23%/96.15); BMYV, beet mild yellowing virus (vg:935287 [https://www.genome.jp/entry/vg:935287]; 95.38%/96.54%); TVDV, tobacco vein distorting virus (vg:6325587 [https://www.genome.jp/entry/vg:6325587]; 50.57%/66.16%); CABYV, cucurbit aphid-borne yellows virus (vg:940449 [https://www.genome.jp/entry/vg:940449]; 26.22%/40.82%); MABYV, melon aphid-borne yellows virus (vg:6369694 [https://www.genome.jp/entry/vg:6369694]; 25.09%/42.70%); PeVYV, pepper vein yellows virus (vg:10192273 [https://www.genome.jp/entry/vg:10192273]; 25.94%/40.23%); PeVYV-5, pepper vein yellows virus 5 (vg:35659779 [https://www.genome.jp/entry/vg:35659779]; 26.22%/40.45%); BVG, barley virus G (vg:27246436 [https://www.genome.jp/entry/vg:27246436]; 26.22%/44.94%); CRLV, carrot red leaf virus (vg:3021801 [https://www.genome.jp/entry/vg:3021801]; 44.64%/61.57%); CCSV, chickpea

chlorotic stunt virus (vg:4187204 [https://www.genome.jp/entry/vg:4187204]; 31.09/48.69%); IYMV-1, ixeridium yellow mottle virus 1 (vg:27111910 [https://www.genome.jp/entry/vg:27111910]; 25.09%/42.80%); PABYV, pepo aphid-borne yellows virus (vg:27924363 [https://www.genome.jp/entry/vg:27924363]; 28.08%/43.08%); SPV-1, strawberry polerovirus 1 (vg:22276102 [https://www.genome.jp/entry/vg:22276102]; 28.47%/47.61%); SABYV, Suakwa aphid-borne yellows virus (vg:13564455 [https://www.genome.jp/entry/vg:13564455]; 25.47%/42.32%); WCMV, white clover mottle virus (vg:30090157 [https://www.genome.jp/entry/vg:30090157]; 30.60%/48.88%); MYDV-RMV, maize yellow dwarf virus RMV (vg:16215725 [https://www.genome.jp/entry/vg:16215725]; 26.87%/46.27%); CYDV-RPS, cereal yellow dwarf virus RPS (vg:1489893; 58.27%/70.30%); CYDV-RPV, cereal yellow dwarf virus RPV (vg:1478313 [https://www.genome.jp/entry/vg:1489893]; 58.05%/70.04%); WYDV-GPV, wheat yellow dwarf virus-GPV (vg:10220411 [https://www.genome.jp/entry/vg:10220411]; 60.61%/70.83%); CPPV-1, cowpea polerovirus 1 (vg:31653049 [https://www.genome.jp/entry/vg:31653049]; 28.68%/47.55%); CPPV-2, cowpea polerovirus 2 (vg:31653057 [https://www.genome.jp/entry/vg:31653057]; 27.17%/43.40%); WLYAV, wheat leaf yellowing-associated virus (vg:33867841 [https://www.genome.jp/entry/vg:33867841]; 28.35%/47.51%); BYDV-GAV, barley yellow dwarf virus GAV (vg:1485846 [https://www.genome.jp/entry/vg:1485846]; 32.21%/48.69%); BYDV-MAV, barley yellow dwarf virus MAV (vg:940436 [https://www.genome.jp/entry/vg:940436]; 33.71%/48.69%); BYDV-PAV, barley yellow dwarf virus PAV (vg:1492000 [https://www.genome.jp/entry/vg:940436]; 32.12%/46.72%); BYDV-PAS, barley yellow dwarf virus PAS (vg:1489885 [https://www.genome.jp/entry/vg:1489885]; 32.12%/46.72%); BYDV-KerII, barley yellow dwarf virus KerII (vg:15842601 [https://www.genome.jp/entry/vg:15842601]; 33.58%/48.68%); RSDAV, rose spring dwarf-associated virus (vg:6369703 [https://www.genome.jp/entry/vg:6369703]; 37.12%/52.27%); CALV, cherry associated luteovirus (vg:30204393

[https://www.genome.jp/entry/vg:30204393]; 41.13%/54.34%); NSPAV, nectarine stem pitting-associated virus (vg:24528016 [https://www.genome.jp/entry/vg:24528016]; 26.44%/44.44%); SDV, soybean dwarf virus (vg:921703 [https://www.genome.jp/entry/vg:921703]; 53.93%/67.42%); BLRV, bean leafroll virus (vg:932046 [https://www.genome.jp/entry/vg:932046]; 57.93%/69.37%); AALV, apple-associated luteovirus (vg:41701548 [https://www.genome.jp/entry/vg:41701548]; 37.16%/55.17%); ALV-1, apple luteovirus 1 (vg:41702098 [https://www.genome.jp/entry/vg:41702098]; 35.93%/54.44%); PALV, peach associated luteovirus (vg:33133630 [https://www.genome.jp/entry/vg:33133630]; 38.87%/53.21%); PEMV-1, pea enation mosaic virus 1 (vg:940255 [https://www.genome.jp/entry/vg:940255]; 28.09%/46.07%); CVEV, citrus vein enation virus (vg:15957166 [https://www.genome.jp/entry/vg:15957166]; 29.74%/44.24%); AEV-1, alfalfa enamovirus 1 (vg:27429657 [https://www.genome.jp/entry/vg:27429657]; 29.00%/43.87%); GEV-1, grapevine enamovirus-1 (vg:32965585 [https://www.genome.jp/entry/vg:32965585]; 20.15%/35.36%).

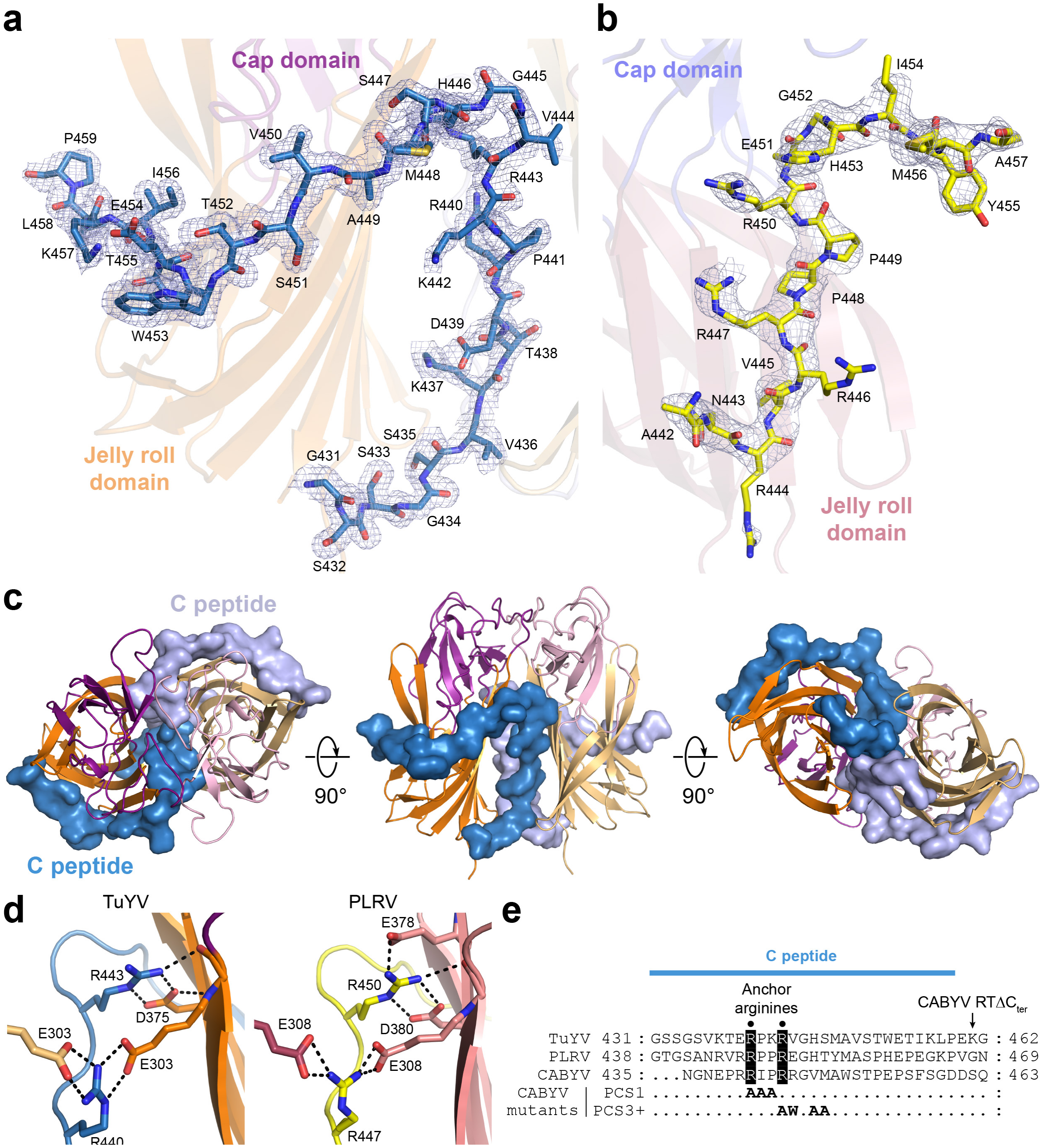

Fig. S6. ^{N}RTD structures resolve clear density for the C peptide. a-b, 2fo-fc electron density (gray mesh) associated with the C peptide in TuYV (a) and PLRV (b) crystal structures contoured to 1σ. The modeled C peptide residues are colored marine and yellow in TuYV (a) and PLRV (b), respectively. c, Segmented surface representation of the C peptides in the context of the TuYV

^{N}RTD dimer. Monomers are colored as in Fig. 2. Top (left), side (middle), and bottom (right) views of the dimer are depicted. d, Anchoring arginines (R440 and R443 in TuYV, left; R447 and R450 in PLRV, right) stabilize the C peptide through a network of hydrogen bonding interactions (dashed black lines). e, C peptide sequences from TuYV, PLRV, and CABYV. Anchoring arginines are marked with dots and highlighted with black boxes. Positions of CABVY C peptide mutants (PCS1 and PCS3+) that prevent RTP incorporation into mature virions[5] are shown below in bold. PCS1 mutant contains alanine substitutions at R441 (anchoring arginine), I442, P443. PCS3+ mutant contains alanine substitutions at R444 (anchoring arginine), V447, and M448 as well as a tryptophan substitution at R445. Arrow denotes the relative position of the CABYV readthrough protein C-terminal truncation that can be normally incorporated into mature virions (RTΔC_{ter}, residue S462)[5].

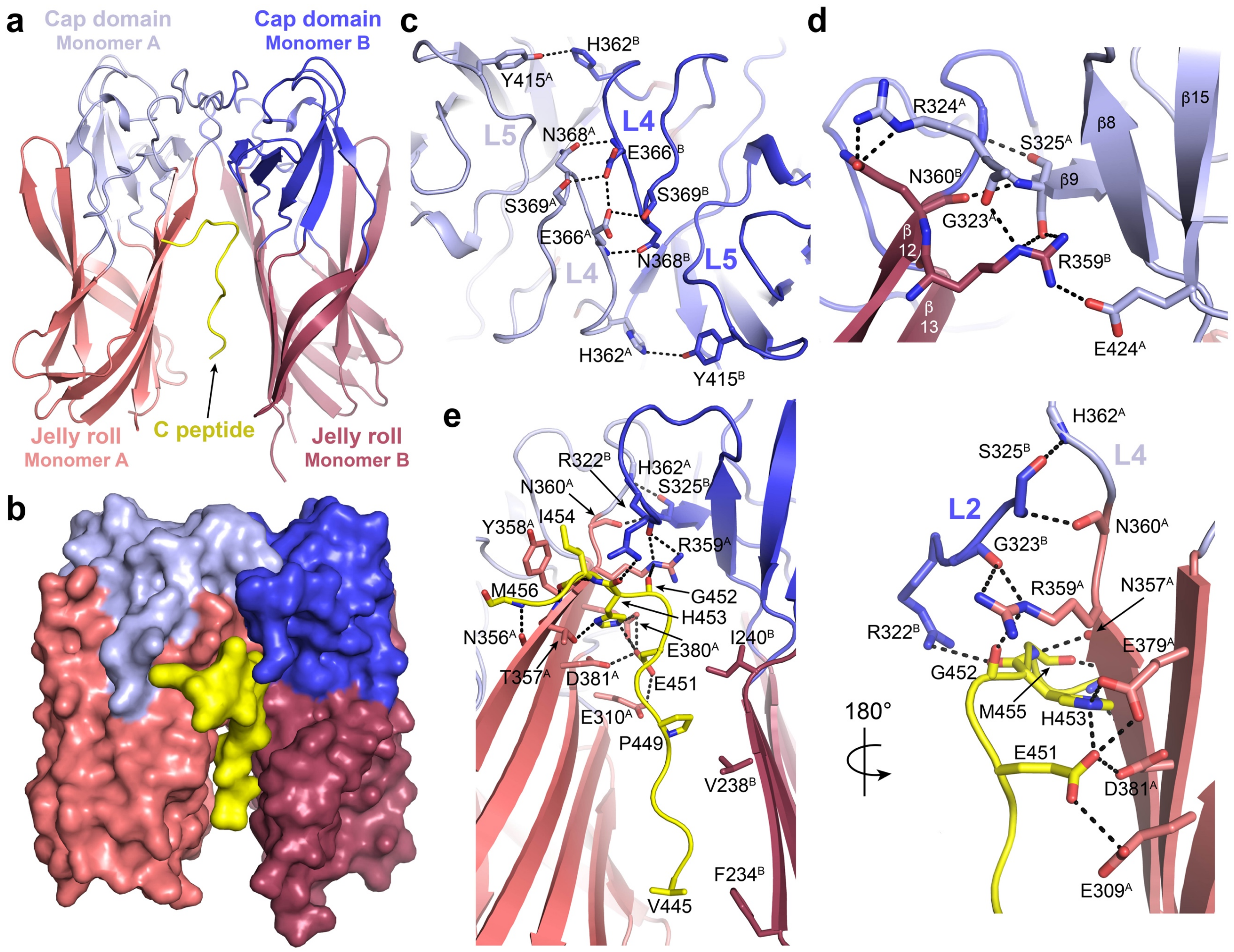

Fig. S7. Architecture of the PLRV ^{N}RTD dimer. a-b, Cartoon (a) and surface (b) representations of the PLRV ^{N}RTD dimer. Individual structural segments are labeled in each monomer and colored as follows: Jelly roll domains, salmon and raspberry; cap domains, light blue and dark blue; C peptide, yellow. c, Slice section through the dimer at the level indicated by the solid line in (a) highlighting stabilizing interactions at the dimer interface. Dashed black lines denote hydrogen bonds. Key residues are labeled with a superscript (A or B) to indicate from which monomer they originate. Secondary structure elements (see Fig. S3) are labeled where applicable. d, Additional stabilizing interactions occurring *in trans* at the upper side of the PLRV dimer. e, C peptide interactions. Residues contributing hydrogen bonding (dashed black lines) and hydrophobic contacts are labeled.

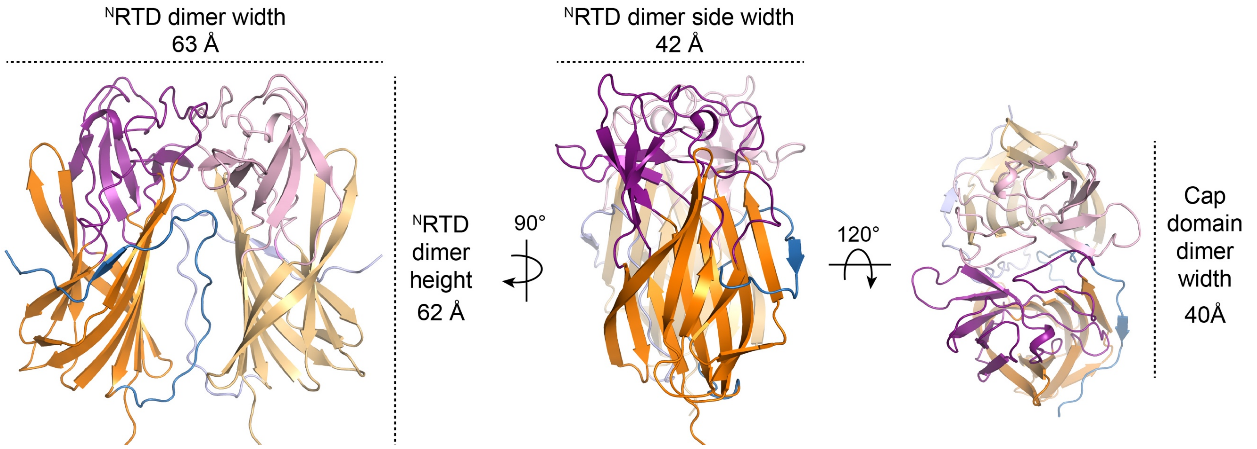

Fig. S8. Dimensions of TuYV NRTD dimer. Dimer width is measured from the ends of the C peptides. Dimer height is measured from the crest of loop 5 down to the ^{N}RTD N-terminus. Dimer side width is measured from the edges of loop two in each monomer. Cap domain dimer width measured from the ends of β3 in each monomer.

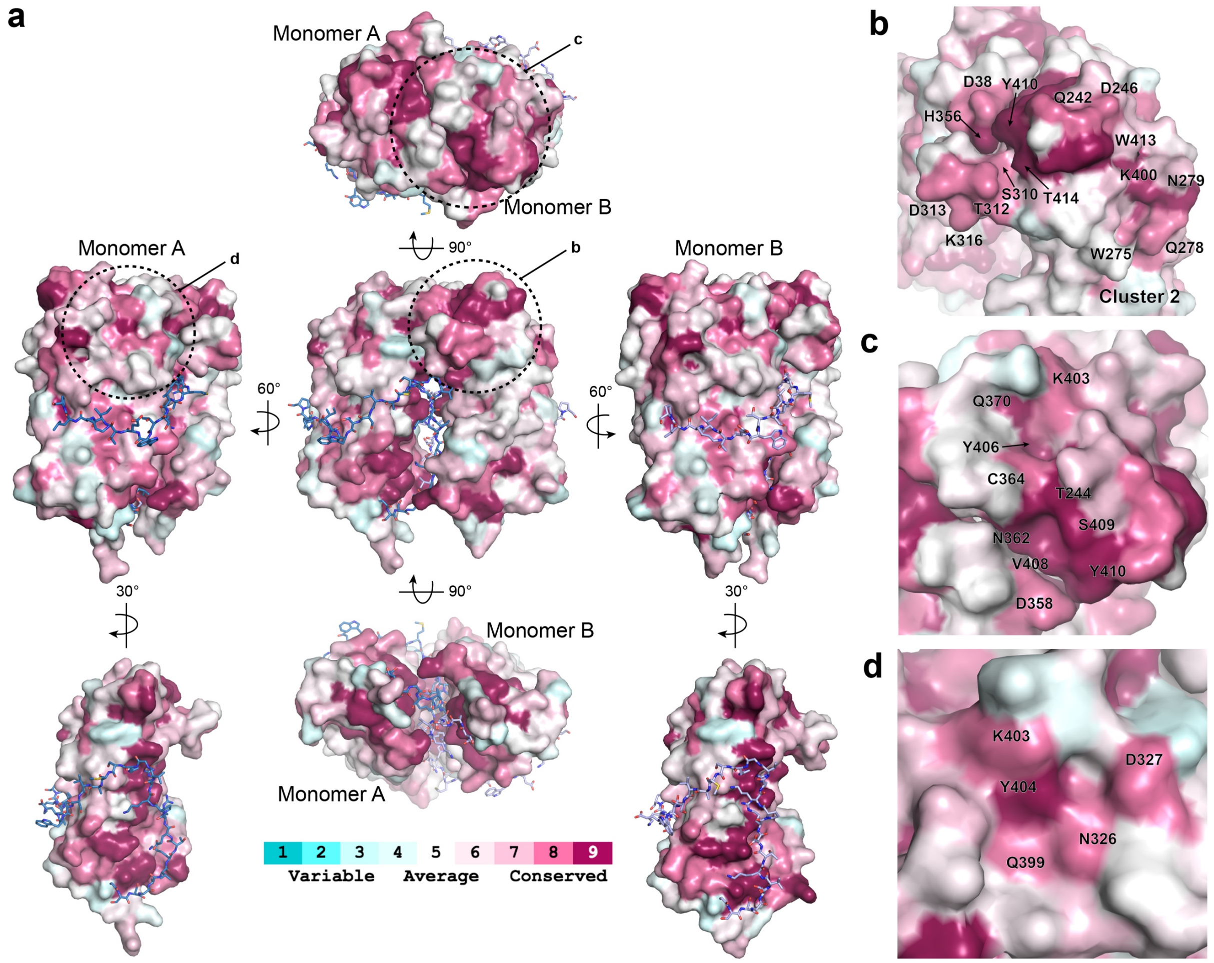

Fig. S9. Structural mapping of polerovirus, enamovirus, and luteovirus sequence conservation. a, Conservation of ^{N}RTD surface exposed residues. Multiple orientations of the TuYV ^{N}RTD dimer (center) and individual monomers (sides, peeled away and rotated relative to the two-symmetry axis) are shown. Coloring reflects sequence conservation across poleroviruses, enamoviruses, and luteoviruses (see legend below) and was generated using the ConSurf server[6] and the alignment in Fig. S5. C peptides are shown as sticks and colored marine (monomer A) and light blue (monomer B). Dashed circles indicate regions highlighted in zoomed views depicted in b, c, and d. b-d, Zoomed views of conserved residue clusters that do not strictly contribute to the dimer interface. Residues are numbered according to TuYV sequence (see Fig. S5).

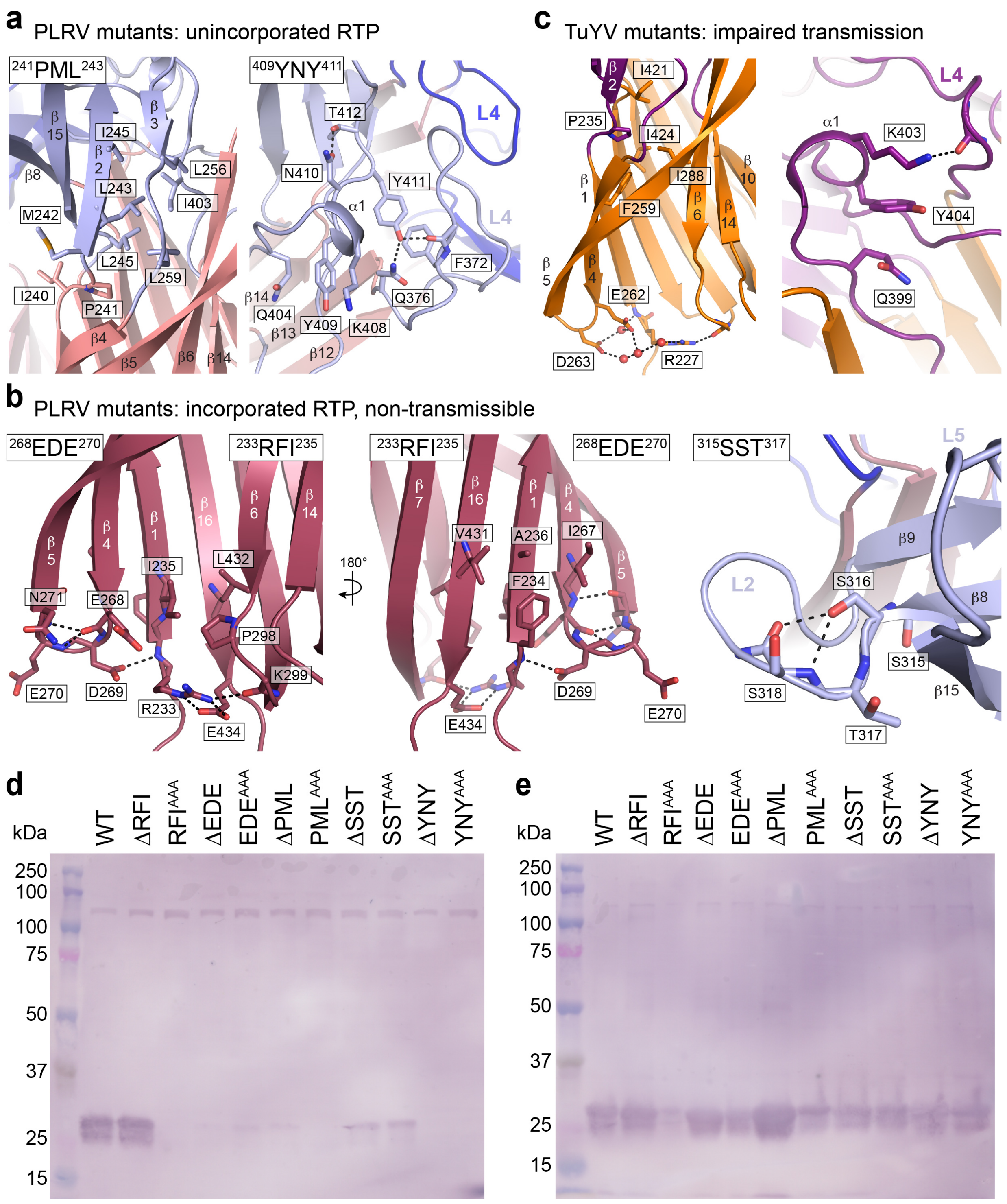

Fig. S10. Location and structural stability of PLRV ^{N}RTD mutants from previous literature. a-b, Location of triplet residue deletions (^{241}PML^{243}, ^{409}YNY^{411}; ^{233}RFI^{235}, ^{268}EDE^{270}, and ^{315}SST^{317}) in the PLRV ^{N}RTD that produced either unincorporated RTP mutants (a) or incorporated RTP

mutants that were nontransmissible[7] (b). Hydrogen bonds (dashed lines) and neighboring side chains involved in hydrophobic interactions are also shown. c, Location of non-transmissible TuYV point mutants (K404 and Y405)[8]. See Figs. 1, S3, S5, S7, and Table S2 for additional info regarding the location and phenotypes of mutated residues in a-c. d-e, Western blot analysis of recombinantly-expressed PLRV ^{N}RTD mutant proteins with either a deletion (Δ) or triple alanine substitution (AAA) mutation. After lysis, insoluble material was pelleted by centrifugation. The supernatant (d) and pellet (e) were both resuspended in lysis buffer, separated by SDS-PAGE gel electrophoresis, and blotted with the anti-^{N}RTD antibody. First lane in both (d) and (e) contains molecular weight markers (kDa). Gels representative of three independently expressed and purified batches of protein.

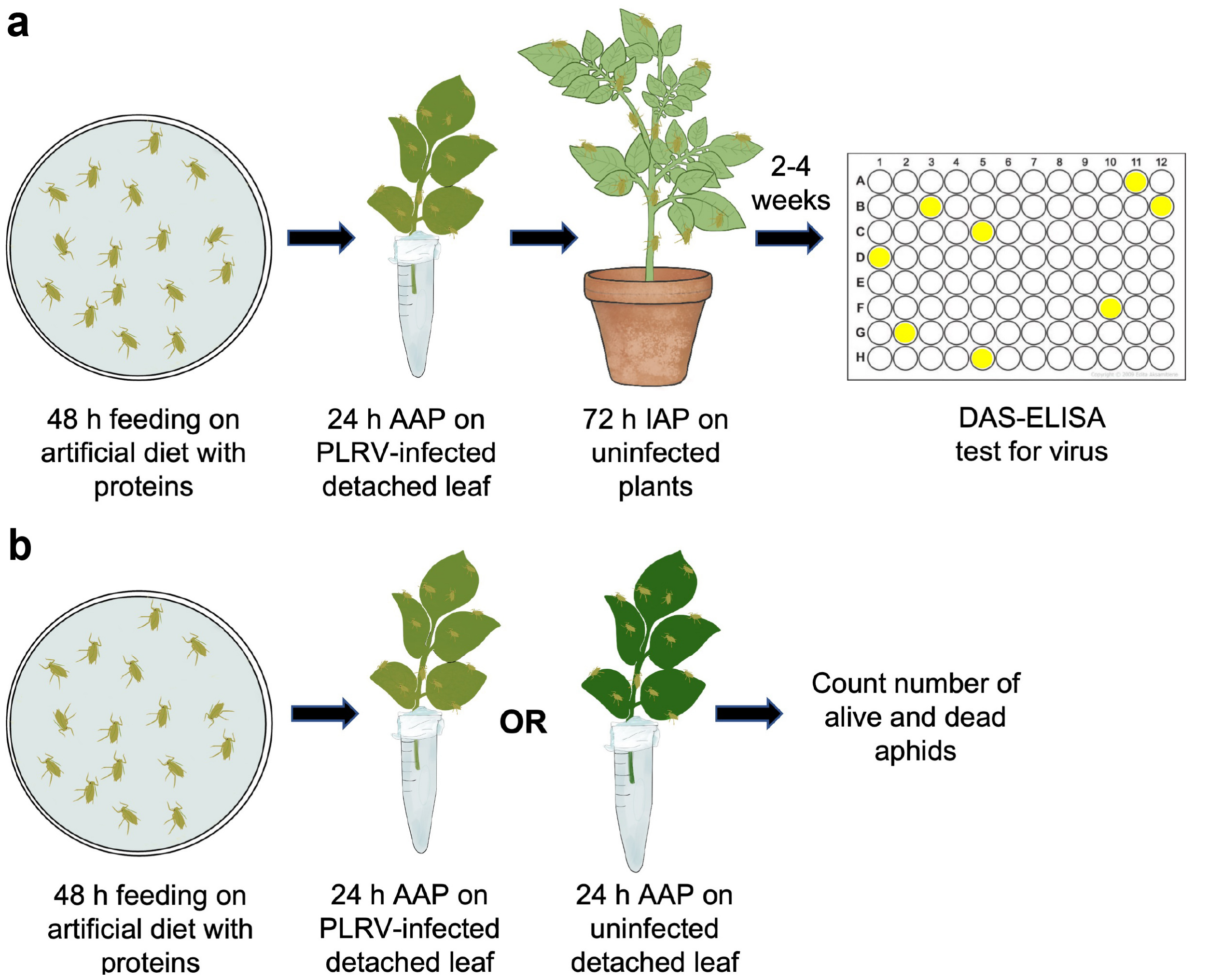

Fig. S11. Experimental design for artificial diet feeding experiments. a, Aphids fed on artificial sucrose diet treatments containing BSA, WT PLRV ^{N}RTD, PLRV ^{N}RTD H321A mutant, or no protein control for 48 hours. Then, aphids were moved to PLRV-infected detached hairy nightshade (HNS) leaves to acquire virus for a 24-hour acquisition access period (AAP). Next, aphids were moved to uninfected potato plants (cv. Red Maria) for a 72-hour an inoculation access period (IAP), 3-5 aphids/plant, 5-16 plants/treatment. After the IAP, aphids were killed by a pesticide application. After several weeks, the inoculated plants are tested for systemic PLRV infection via DAS-ELISA. b, Age-synchronized *M. persicae* aphids were allowed to feed on 0.1 mg/mL of BSA, WT PLRV ^{N}RTD or PLRV ^{N}RTD mutants (H321A, E366A, H371A, E374A or cluster mutant containing N368A, C370A, Y411A substitutions) in artificial diet for 48 hours before

being moved to a PLRV-infected or uninfected detached HNS leaf. After 24 hours on the HNS leaves, mortality of *M. persicae* aphids was tallied for each treatment.

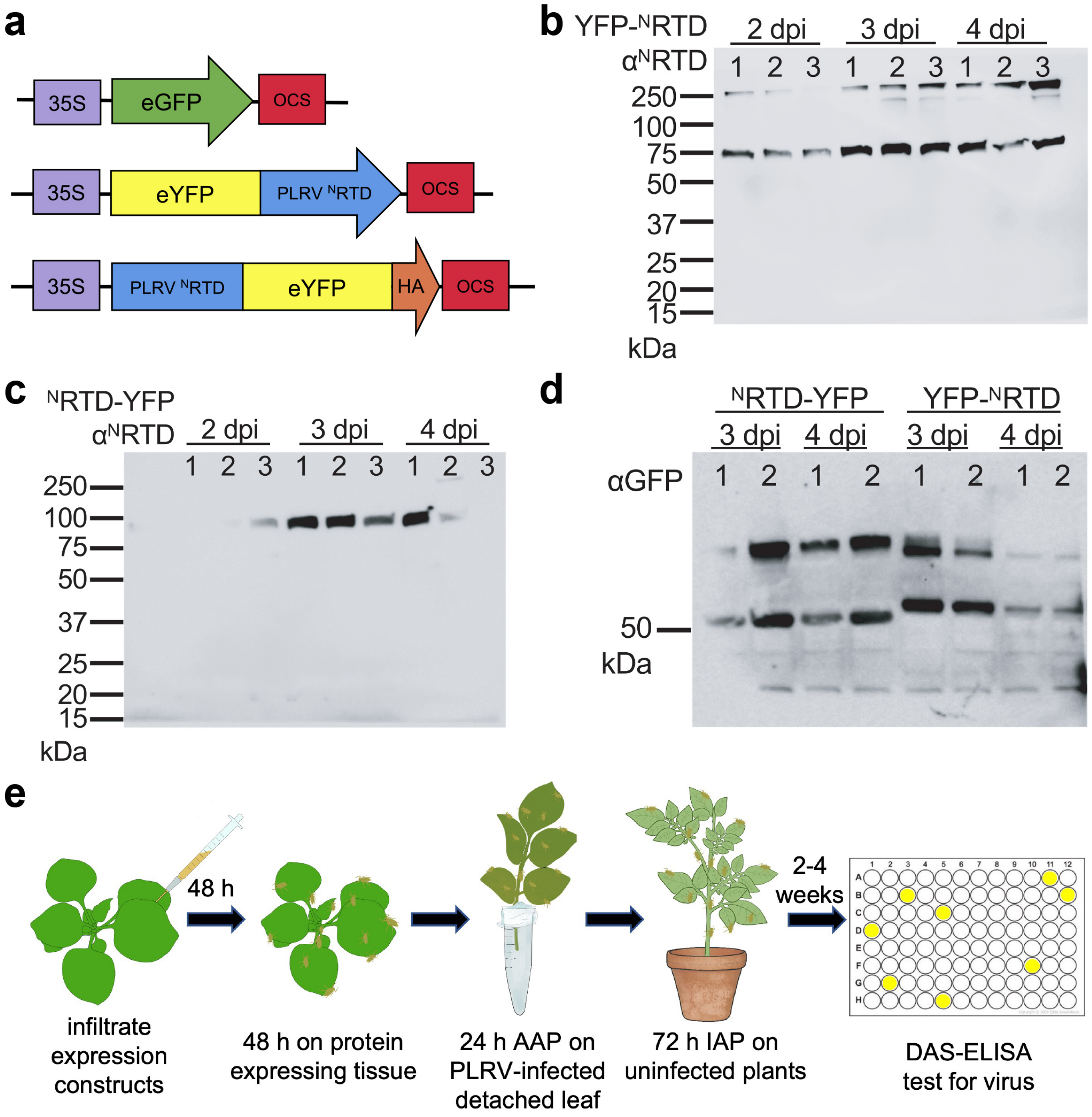

Fig. S12. Transient *in planta* delivery of the PLRV ^{N}RTD to aphids. a, Construct design for transient *in planta* expression of the PLRV ^{N}RTD. b-c, Western blot analysis of expression tests of YFP-^{N}RTD (b) and ^{N}RTD-YFP (c) blotted with the anti-^{N}RTD antibody. d, Samples from the same expression tests in (b) and (c) blotted with an anti-GFP antibody. *In planta* expression tests and subsequent analysis via western blot (b-d) was repeated independently twice for each construct. e, Experimental design for the transient *in planta* delivery experiments. *N. benthamiana*

plants were infiltrated with transient expression constructs. At 2 days post inoculation (dpi) aphids fed on protein-expressing tissue for 48 hours. Then, *M. persicae* aphids were moved to PLRV-infected detached hairy nightshade leaves to acquire virus for a 24-hour acquisition access period (AAP). Next, aphids were moved to uninfected potato plants (cv. Red Maria) for a 72-hour an inoculation access period (IAP), 5 aphids/plant, 10-15 plants/treatment. After the IAP, aphids were killed by a pesticide application. After several weeks, the inoculated plants are tested for systemic PLRV infection via DAS-ELISA. n = 37 for all treatments.

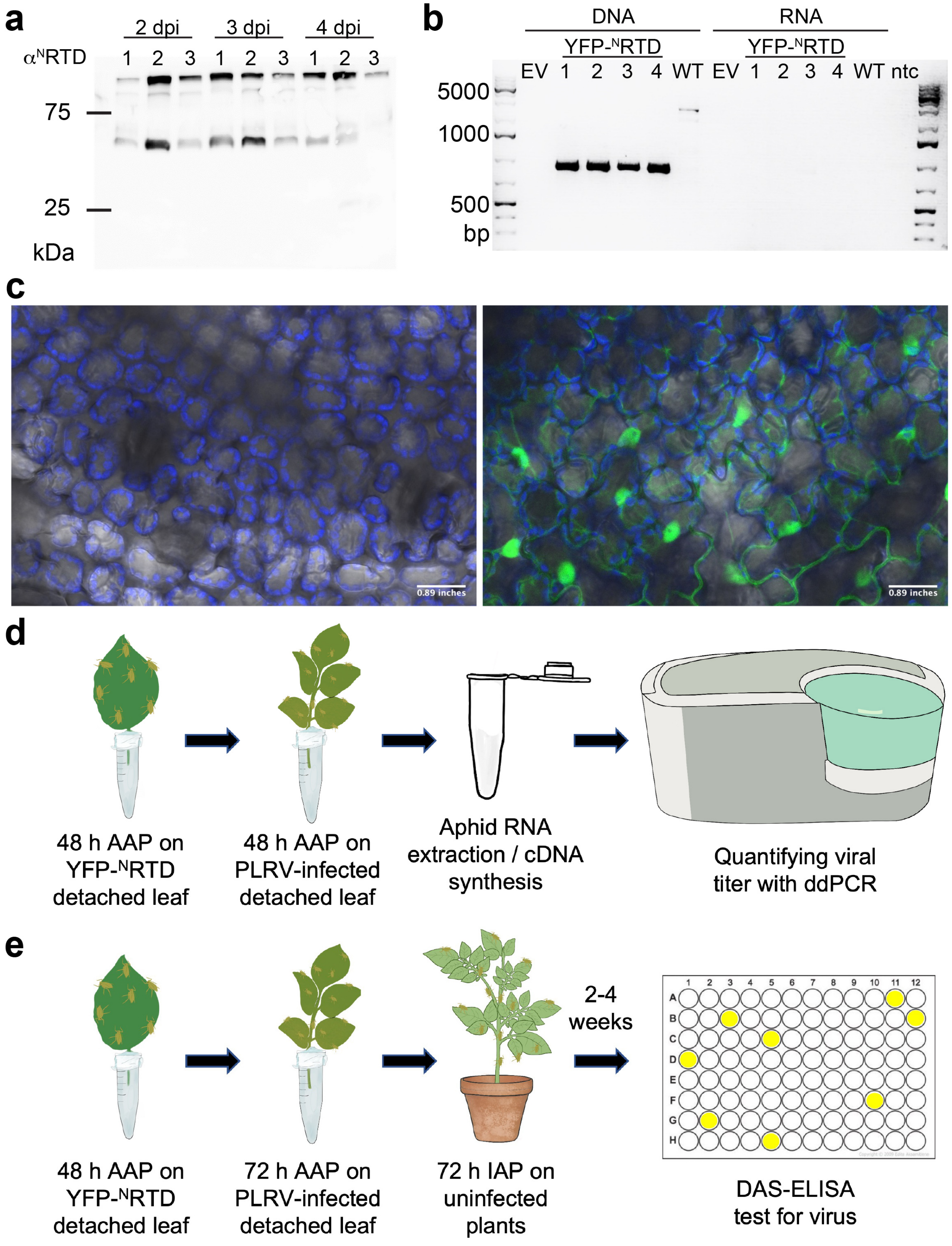
a
2 dpi
3 dpi
4 dpi
α^NRTD
1 2 3 1 2 3 1 2 3
75
25
kDa
b
DNA
RNA
YFP-^NRTD
YFP-^NRTD
EV 1 2 3 4 WT EV 1 2 3 4 WT ntc
5000
1000
500
bp
c
d
48 h AAP on YFP-^NRTD detached leaf
48 h AAP on PLRV-infected detached leaf
Aphid RNA extraction / cDNA synthesis
Quantifying viral titer with ddPCR
e
48 h AAP on YFP-^NRTD detached leaf
72 h AAP on PLRV-infected detached leaf
72 h IAP on uninfected plants
2-4 weeks
DAS-ELISA test for virus

Fig. S13. Transgenic potato delivery of the ^{N}RTD. a, Western blot analysis of YFP-^{N}RTD transiently expressed in *N. benthamiana* blotted with the ^{N}RTD antibody. This experiment was conducted once to confirm construct expression in *N. benthamiana* before transformation of potato. b, RT-PCR of transgenic potatoes as well as WT, empty vector (EV) and non-template (ntc) controls using cDNA or RNA as template. RT-PCR analysis was repeated independently at least three times. c, Visualization of YFP-^{N}RTD fluorescence (green) via laser scanning confocal microscopy. Empty vector transgenic plants were used a control (left). Blue is chloroplast autofluorescence. Visualization of YFP in transgenic and control potato plants was conducted independently three times with similar results. d-e, Experimental design for testing aphid acquisition (d) and transmission (e) of PLRV after transgenic delivery of the ^{N}RTD. Aphids were exposed to YFP-^{N}RTD transgenic leaves for 48 hours before being moved to PLRV-infected detached hairy nightshade leaves to acquire virus for a 48-hour acquisition access period (AAP). Next, in acquisition experiments (d), aphids were flash frozen and later total RNA was extracted and cDNA synthesized. Copies of PLRV in aphids was quantified via digital drop PCR. In transmission experiments (e), after acquisition aphids were moved to uninfected potato plants (cv. Red Maria) for a 72-hour acquisition access period (IAP), 5 aphids/plant, 11-45 plants/treatment. After the IAP, aphids were killed by a pesticide application. After several weeks, the inoculated plants are tested for systemic PLRV infection via DAS-ELISA.

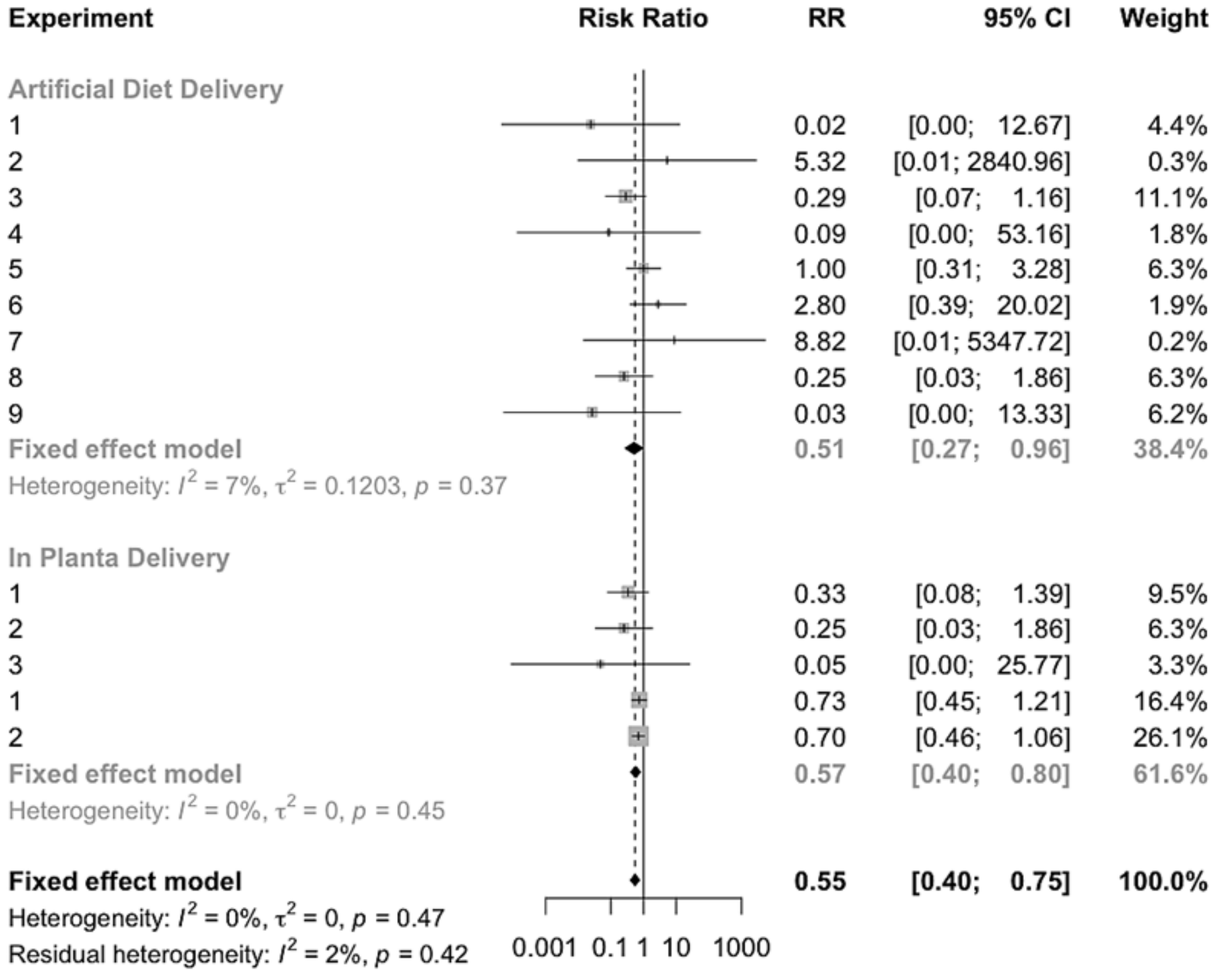

Fig. S14. Meta-analysis and forest plot of all WT PLRV ^{N}RTD trials. Forest plot showing metanalysis of various trials of WT ^{N}RTD feeding transmission assays using a fixed effects model. Graphed is the risk ratio (RR) of a plant becoming infected with PLRV after pre-exposure of aphids to the WT ^{N}RTD as compared to the control, grouped by whether the ^{N}RTD was delivered via artificial diet or *in planta* (transient 1-3 or transgenic 1-2). The vertical axis (x =1) represents the line of no effect. Values to the left of this line indicate a reduced chance of PLRV infection. Vertical dashes represent the point estimate of the risk ratio from each individual experiment (also shown in the "RR" column). The size of the gray box surrounding the vertical dash corresponds to the relative weight of that experiment in the analysis (also depicted numerically in the column "Weight"), which is correlated with how many plants became infected in that experiment.

Horizontal lines (“whiskers”) represent the 95% confidence interval (95% CI) of the risk ratio in each study (also shown numerically under the “95% CI” column). The center of the diamond represents the point estimate of the risk ratio for each subgroup or all experiments pooled (bottom), with the width of the diamond representing the 95%CIThe vertical dashed line shows the position of the pooled risk ratio for all experiments. Shown at the bottom are two measures of heterogeneity between experiments (Higgins and Thompson’s I^2 and heterogeneity variance, t^2) and the *P*-value of a one-sided Cochran’s *Q* test for subgroup differences. Sample size is $n = 9$ independent replicates of the artificial diet delivery experiment and $n = 5$ independent replicates of *in planta* expression experiments. Full results for each experiment are included in Supplementary Tables S3, S5, and S7.

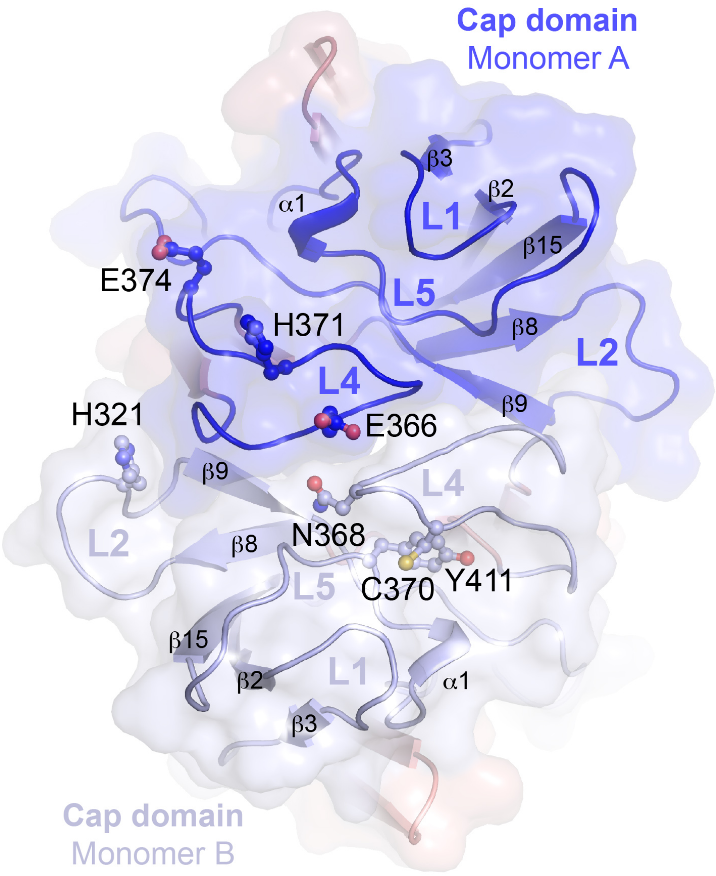

Fig. S15. Location of PLRV cap domain mutants. Top view of PLRV ^{N}RTD dimer illustrating the positions of the cap domain residues H321, E366, H371, E374, N368, C370, and Y411 that were mutated and tested in feeding assays. Secondary structure elements and cap domain loops are labeled. The PLRV ^{N}RTD dimer is shown in the same orientation as the TuYV ^{N}RTD dimer in Fig. 5a. See Figs. 4a and 5b for the phenotypes associated with the cap domain alanine mutants.

Table S1. Data collection and refinement statistics*.

	PLRV ^{N}RTD	TuYV ^{N}RTD
**Data collection**		
Space group	$P2_122_1$	$P2_12_12_1$
Cell dimensions		
a, *b*, *c* (Å)	63.23, 65.15, 109.68	46.46, 74.86, 130.78
α, β, γ(°)	90, 90, 90	90, 90, 90
Resolution (Å)	56.01-2.22 (2.29-2.22)	64.97-1.53 (1.55-1.53)
R_{merge}	0.01 (0.85)	0.07 (0.73)
$I / \sigma I$	10.5 (0.8)	20.5 (2.2)
Completeness (%)	96.8 (75.2)	99.5 (93.1)
Redundancy	5.1 (2.0)	6.7 (6.3)
**Refinement**		
Resolution (Å)	2.22	1.53
No. reflections	22407	69703
R_{work} / R_{free}	20.1/24.6	17.4/19.6
No. atoms		
Protein	3437	3822
Ligand/ion	5	0
Water	16	587
B-factors		
Protein	57.6	23.6
Ligand/ion	54.1	-
Water	45.7	33.6
R.m.s deviations		
Bond lengths (Å)	0.01	0.01
Bond angles (°)	1.3	1.3

*Values in parentheses are for highest-resolution shell. Each structure was determined from a single crystal.

Table S2. Summary of PLRV and TuYV ^{N}RTD mutation reverse genetic studies from the literature

Name	Residue	Position	Mutation	Conservation[a]	RTP Incorporated[b]	Plant Systemic Infection	Aphid Transmission	Comments
**Peter *et al.* 2008 - potato leafroll virus**								
	R	233	deletion	identical				
∆RFI*	F	234	deletion	identical	Yes	Like WT	No	
	I	235	deletion	similar				
	S	315	deletion	identical				
∆SST*	S	316	deletion	not conserved	Yes	Like WT	No	
	T	317	deletion	not conserved				
	E	268	deletion	identical				
∆EDE*	D	269	deletion	similar	Yes	Like WT	No	
	E	270	deletion	identical				
	P	241	deletion	identical				
∆PML*	M	242	deletion	not conserved	No	-	No	This motif is PVT in TuYV
	L	243	deletion	not conserved				
	Q	284	deletion	similar				
∆QSS	S	285	deletion	similar	No	Delayed compared to WT	No	
	S	286	deletion	not conserved				
	K	299	deletion	not conserved				
∆KGQ	G	300	deletion	identical	No	-	No	
	Q	301	deletion	not conserved				
	I	329	deletion	identical				
∆IAY	A	330	deletion	identical	No	-	No	
	Y	331	deletion	identical				
∆GHPE	G	361	deletion	identical	No	Delayed compared to WT	No	
	H	362	deletion	identical				

	P	363	deletion	not conserved				
	E	364	deletion	similar				
	E	379	deletion	identical				
ΔERD	R	380	deletion	similar	No	Delayed compared to WT	No	
	D	381	deletion	identical				
	Y	409	deletion	identical				
ΔYNY*	N	410	deletion	identical	No	-	No	
	Y	411	deletion	identical				
	S	414	deletion	identical				
ΔSYG	Y	415	deletion	identical	No	-	No	
	G	416	deletion	identical				
	D	420	deletion	similar				
DDRD	R	421	deletion	similar	No	-	No	
	D	422	deletion	not conserved				
	L	432	deletion	similar				
DLDE	D	433	deletion	identical	No	-	No	
	E	434	deletion	identical				
**Brault *et al.* 2000 - turnip yellows virus**								
R227A	R	227	A	identical	Yes	Accumulation slightly lower than WT	Reduced	Same R as in ΔRFI
	Compensatory Second Site Mutation[c]							
	P	235	L	identical	Yes	Like WT	Like WT	Same P as in ΔPML
ED^{AA}	E	262	A	identical	Yes	Accumulation slightly lower than WT	Only after microinjection	Same ED as in ΔEDE
	D	263	A	similar				
	Compensatory Second Site Mutation[c]							
	P	235	L	identical	Yes	Like WT	Like WT	Same P as in ΔPML
KD^{AA}	K	316	A	similar	Yes	Accumulation slightly lower than WT	Like WT	Just downstream of the SST motif in TuYV
	D	317	A	not conserved				

KY^{AD}	K	403	A	identical	Yes	Accumulation much lower than WT	Reduced, improved by microinjection	Same Y as in ΔYNY
	Y	404	D	identical				
	Revertant[c]							
	Y	404	Y	identical	Yes	Like WT	Like WT	Same Y as in ΔYNY
	Pseudorevertants[c]							
	Y	404	F	identical	Yes	-	Like WT	Same Y as in ΔYNY
	Y	404	N	identical	Yes	-	-	Same Y as in ΔYNY
DE^{AA}	D	428	A	identical	Yes	Accumulation slightly lower than WT	Like WT	Same DE as in ΔLDE
	E	429	A	identical				
P235L	P	235	L	identical	Yes	Like WT	Like WT	Same as second site mutation, but in WT

[a] Degree of conservation of the residue across the *Polerovirus, Enamovirus* and *Luteovirus* genera
[b] Indicating whether or not this mutation prevented proper incorporation of the RTP into the assembled virion
[c] In Brault *et al.* 2000, the authors tracked if these mutant forms of the virus developed second site mutations, reverted to the WT residue, or pseudoreverted to another residue and tested those mutant viruses as well.
* These mutations were tested in our purified PLRV ^{N}RTD solubility test.
- Not tested in the study
Abbreviations: PLRV, potato leafroll virus; TuYV, turnip yellows virus; RTP, readthrough protein; WT, wild type

Table S3. PLRV transmission by *M. persicae* aphids after artificial diet delivery of WT PLRV ^{N}RTD and H321A.

Exp	No Protein Control		Conc	BSA		WT PLRV ^{N}RTD		H321A	
	Infected/ Total	Transmission Efficiency[a]		Infected/ Total	Transmission Efficiency[a]	Infected/ Total	Transmission Efficiency[a]	Infected/ Total	Transmission Efficiency[a]
1[b]	2/6	33%	0.1 mg/mL	4/6	67%	0/6	0%	-	-
			1 mg/mL	3/6	50%	0/6	0%	-	-
2[b]	0/12	0%	0.1 mg/mL	4/12	33%	1/12	8%	-	-
			1 mg/mL	2/5	40%	1/12	8%	-	-
3	7/15	47%	0.1 mg/mL	7/15	47%	2/15	13%	-	-
4	1/15	7%	0.1 mg/mL	5/15	33%	0/16	0%	-	-
5	4/15	27%	0.1 mg/mL	4/15	27%	4/15	27%	-	-
6	1/7	14%	0.1 mg/mL	7/10	70%	1/10	10%	4/10	40%
7	0/8	0%	0.1 mg/mL	2/9	22%	1/10	10%	2/10	20%
8[c]	4/10	40%	0.1 mg/mL	1/10	10%	1/10	10%	0/10	0%
9	4/13	31%	0.1 mg/mL	2/13	15%	0/12	0%	2/13	15%
Total	23/101	23%		41/116	35%	11/124	9%	8/43	19%

[a] Percent of plants that become systemically infected with PLRV after inoculation by aphids exposed to no protein control, BSA, or PLRV ^{N}RTD. Aphids were exposed to diets for 48 h, followed by an acquisition access period and 72-h inoculation access period. Plants were tested for virus via ELISA 4 weeks post inoculation.
[b] Plants in experiments 1 and 2 were inoculated by 3 aphids/plants. All subsequent experiments used 5 aphids/plant.
[c] Aphids were given a 48-h acquisition access period in experiment 8. The acquisition access period was 24-h in all other experiments.
- This treatment not tested in this experiment.
Abbreviations: PLRV, potato leafroll virus; WT, wild type; Exp, Experiment; Conc, concentration; BSA, bovine serum albumin

Table S4. Logistic regression analysis of PLRV transmission by *M. persicae* aphids after artificial diet delivery of PLRV ^{N}RTD and H321A.

Predictor[a]	β	*SE* β	Wald *z*	*df*	*Pr* >| *z* |
Intercept	- 0.786	± 0.483	- 1.627	1	0.104
Treatment[b] (vs. no protein control)					
BSA	0.627	± 0.318	1.971	1	0.049 *
WT PLRV ^{N}RTD	- 1.087	± 0.464	- 2.342	1	0.019 *
H321A	- 0.121	± 0.524	- 0.232		0.817
Exp[c] (vs. experiment 1)					
2	- 0.837	± 0.578	- 1.446	1	0.148
3	0.266	± 0.535	0.498	1	0.619
4	- 1.114	± 0.616	- 1.809	1	0.071
5	- 0.190	± 0.550	- 0.346	1	0.730
6	0.371	± 0.611	0.607	1	0.544
7	- 1.166	± 0.712	- 1.639	1	0.101
8	- 1.028	± 0.670	- 1.534	1	0.125
9	- 0.743	± 0.605	- 1.228	1	0.220

Test	H_o	χ^2	*df*	$P > \chi^2$
Removing Exp[c] from Model	$\beta_{Exp} = 0$	17.78	8	0.023 *
BSA vs. WT ^{N}RTD	$\beta_{BSA} = \beta_{WT\ PLRV\ NRTD}$	15.00	1	< 0.001 ***
BSA vs. H321A	$\beta_{BSA} = \beta_{H321A}$	2.20	1	0.140
Overall Model Evaluation				
Likelihood Ratio Test	$\beta_{BSA} = \beta_{WT\ PLRV\ NRTD} = 0$	36.58	11	< 0.001 ***
Wald Test	$\beta_{BSA} = \beta_{WT\ PLRV\ NRTD} = 0$	31.5	11	< 0.001 ***
Goodness-of-Fit Test	the model fits	334.42	328	0.392

[a] Model output is the categorical variable "InfectionState" with levels 0 = uninfected and 1 = PLRV-infected indicating whether the inoculated plant became systemically infected

[b] "Treatment" is a categorical variable with levels 0 = no protein control, 1 = BSA, 2 = PLRV ^{N}RTD, 3 = H321A

[c] "Exp" is a categorical variable representing the different trials of the experiment

Abbreviations: PLRV, potato leafroll virus; *SE*, standard error; *df,* degrees of freedom; BSA, bovine serum albumin; WT, wild type; H_o, null hypothesis

Table S5. PLRV transmission by *M. persicae* aphids after transient *in planta* delivery of PLRV ^{N}RTD

Exp	Uninfiltrated Control		GFP		YFP-^{N}RTD		^{N}RTD-YFP	
	Infected/ Total	Transmission Efficiency[a]	Infected/ Total	Transmission Efficiency[a]	Infected/ Total	Transmission Efficiency[a]	Infected/ Total	Transmission Efficiency[a]
1	6/15	40%	7/15	47%	2/15	13%	6/15	40%
2	4/10	40%	2/10	20%	1/10	10%	0/10	0%
3	2/12	17%	1/12	8%	0/12	0%	2/12	17%
Total	12/37	32%	10/37	27%	3/37	8%	8/37	22%

[a] Percent of plants that become systemically infected with PLRV after inoculation by aphids exposed to uninfiltrated control, GFP, YFP-^{N}RTD or ^{N}RTD-YFP. *N. benthamiana* plants were infiltrated with transiently expression constructs. 2 days post inoculation, aphids were placed on infiltrated tissue for 48 h, followed by a 24-hour acquisition access period and 72-h inoculation access period with 5 aphids/plants. Plants were tested for virus via ELISA 4 weeks post inoculation.

Abbreviations: PLRV, potato leafroll virus; Exp, Experiment; GFP, green fluorescent protein; YFP, yellow fluorescent protein

Table S6. Logistic regression analysis of PLRV transmission by *M. persicae* aphids after transient *in planta* delivery of PLRV ^{N}RTD.

Predictor[a]	β	*SE* β	Wald *z*	*df*	*Pr* >| *z* |
Intercept	0.076	± 0.430	0.176	1	0.860
Treatment[b] (vs. uninfiltrated control)					
35S:GFP	- 0.620	± 0.563	- 1.100	1	0.271
35S:YFP-^{N}RTD	- 1.838	± 0.724	- 2.538	1	0.011 *
35S:^{N}RTD-YFP	- 0.620	± 0.563	- 1.100	1	0.271
Exp[c] (vs. experiment 1)					
2	- 1.403	± 0.564	- 2.486	1	0.013 *
3	- 1.615	± 0.559	- 2.891	1	0.004 **

Test	H_o	χ^2	*df*	$P > \chi^2$
Removing Exp[c] from Model	$\beta_{Exp} = 0$	21.36	34	0.002 **
GFP vs. YFP-^{N}RTD	$\beta_{GFP} = \beta_{YFP\text{-}NRTD}$	2.70	1	0.100
GFP vs. ^{N}RTD-YFP	$\beta_{GFP} = \beta_{NRTD\text{-}YFP}$	< 0.001	1	1.000
YFP-^{N}RTD vs. ^{N}RTD-YFP	$\beta_{YFP\text{-}NRTD} = \beta_{NRTD\text{-}YFP}$	2.70	1	0.100
Overall Model Evaluation				
Likelihood Ratio Test	all $\beta_i = 0$	19.83	5	0.001 **
Wald Test	all $\beta_i = 0$	16.30	5	0.006 **
Goodness-of-Fit Test	the model fits	132.09	142	0.713

[a] Model output is the categorical variable “InfectionState” with levels 0 = uninfected and 1 = PLRV-infected indicating whether the inoculated plant became systemically infected

[b] “Treatment” is a categorical variable with levels 0 = uninfiltrated control, 1 = 35S:GFP, 2 = 35S:YFP-^{N}RTD, 3 = 35S:^{N}RTD-YFP

[c] “Exp” is a categorical variable representing the different trials of the experiment

Abbreviations: *SE*, standard error; *df,* degrees of freedom; H_o, null hypothesis; GFP, green fluorescent protein; YFP, yellow fluorescent protein

Table S7. PLRV transmission by *M. persicae* aphids after transgenic potato delivery of PLRV ^{N}RTD

Exp	Empty Vector Control		PLRV ^{N}RTD Transgenic	
	Infected/ Total	Transmission Efficiency[a]	Infected/ Total	Transmission Efficiency[a]
1	9/11	82%	9/15	60%
2	11/15	73%	23/45[b]	20%
Total	20/26	77%	32/60	53%

[a] Percent of plants that become systemically infected with PLRV after inoculation by aphids exposed to empty vector control of PLRV N-RTD expressing transgenic potato plants. Aphids were placed on potato leaves for 48 h, followed by a 24-hour acquisition access period and 72-h inoculation access period with 5 aphids/plants. Plants were tested for virus via ELISA 4 weeks post inoculation.

[b] This experiment used three different independent potato transgenics, 15 plants inoculated per transgenic plant, for a total of 45 plants inoculated.

Abbreviations: PLRV, potato leafroll virus*;* Exp, Experiment

Table S8. Logistic regression analysis of PLRV transmission by *M. persicae* aphids after transgenic potato delivery of PLRV ^{N}RTD.

Predictor[a]	β	*SE* β	Wald *z*	*df*	*P*r >| *z* |
Intercept	1.200	± 0.466	2.587	1	0.010 *
Treatment[b] (vs. empty vector control)					
Transgenic PLRV ^{N}RTD	- 1.070	± 0.533	- 2.010	1	0.044 *

Test	H_o	χ^2	*df*	*P* > χ^2
Removing Exp[c] from Model	$\beta_{Exp} = 0$	1.39	1	0.435
Overall Model Evaluation				
Likelihood Ratio Test	$\beta_{EVC} = \beta_{TG\ PLRV\ NRTD} = 0$	4.43	1	0.035 *
Wald Test	$\beta_{EVC} = \beta_{TG\ PLRV\ NRTD} = 0$	4.00	1	0.044 *
Goodness-of-Fit Test	the model fits	111	84	0.026 *

[a] Model output is the categorical variable “InfectionState” with levels 0 = uninfected and 1 = PLRV-infected indicating whether the inoculated plant became systemically infected.
[b] “Treatment” is a categorical variable with levels 0 = empty vector control, 1 = transgenic PLRV ^{N}RTD
[c] “Exp” is a categorical variable representing the different trials of the experiment.
Abbreviations: *SE*, standard error; *df,* degrees of freedom; H_o, null hypothesis; EVC = empty vector control; TG = transgenic.

Table S9. Mortality of *M. persicae* aphids after artificial diet delivery of PLRV ^{N}RTD mutants.

		Uninfected Leaf			PLRV-infected leaf		
Treatment[a]	Exp	Dead	Alive	Mortality[b]	Dead	Alive	Mortality[b]
No Protein Control	1	0	14	0%	1	49	2%
	2	2	38	5%	1	39	3%
	3	1	48	2%	3	48	6%
BSA	1	-	-	-	2	48	4%
	2	1	43	2%	0	50	0%
	3	0	50	0%	2	46	4%
WT PLRV ^{N}RTD	1	4	15	21%	9	39	19%
	2	3	38	7%	1	36	3%
	3	4	47	8%	3	48	6%
H321A	2	1	41	2%	1	42	2%
	3	4	46	8%	1	47	2%
E366A	1	10	7	59%	44	4	92%
	2	25	16	61%	33	9	79%
H371A	1	31	8	79%	45	3	94%
	2	37	3	93%	41	0	100%
	3	45	5	90%	42	4	91%
E374A	2	37	1	97%	28	0	100%
	3	50	2	96%	41	2	95%
cluster	1	26	4	87%	48	0	100%
	2	39	2	95%	40	0	100%
	3	46	1	98%	47	1	98%

[a] *M. persicae* aphids were exposed to proteins in artificial diet (0.1 mg/mL) for 48 hours before being moved to an uninfected or PLRV-infected detached leaf.
[b] Mortality after 24 hours on detached leaves
-This treatment not tested in this experiment
Abbreviations: PLRV, potato leafroll virus; Exp, Experiment; WT, wild type; BSA, bovine serum albumin

Table S10. Quasibinomial regression analysis of *M. persicae* mortality after artificial diet delivery of PLRV ^{N}RTD mutants.

Predictor[a]	β	*SE* β	*t*	*df*	*P*r >| *t* |
Intercept	- 3.906	± 0.528	- 7.391	1	< 0.001 ***
Treatment[b] (vs. no protein control)					
BSA	- 0.403	± 0.812	2.437	1	0.623
WT PLRV ^{N}RTD	1.385	± 0.568	2.437	1	0.021 *
H321A	0.251	± 0.738	0.340	1	0.736
E366A	4.560	± 0.553	8.242	1	< 0.001 ***
H371A	5.947	± 0.585	10.166	1	< 0.001 ***
E374A	7.172	± 0.802	8.938	1	< 0.001 ***
cluster	6.866	± 0.671	10.233	1	< 0.001 ***
InfectedLeaf[c] (vs. uninfected leaf)	0.684	± 0.266	2.576	1	0.015 *

Test	H_o	χ^2	*df*	$P > \chi^2$
Removing Exp[d] from Model	$\beta_{Exp} = 0$	0.009	3	0.996
Overall Model Evaluation				
Likelihood Ratio Test	all $\beta_i = 0$	337.5	7	< 0.001 ***
Wald Test	all $b_{\iota} = 0$	37.807	8	< 0.001 ***
Goodness-of-Fit Test	the model fits	1.4051	32	1.000

[a] Model output is the numerical variable “PercentDead” with values between 0 and 1 indicating what proportion of aphids died

[b] “Treatment” is a categorical variable with levels 0 = no protein control, 1 = BSA, 2 = WT PLRV ^{N}RTD, 3 = H321A, 4 = E366A, 5= H371A, 6= E374A, 8 = cluster

[c] “InfectedLeaf” is a categorical variable with levels 0 = uninfected leaf, 1= PLRV-infected leaf

[d] “Exp” is a categorical variable representing the different trials of the experiment

Abbreviations: *SE*, standard error; *df,* degrees of freedom; WT, wild type; BSA, bovine serum albumin, H_o, null hypothesis

SUPPLEMENTARY REFERENCES

1 Byrne, M. J. *et al.* Combining transient expression and cryo-EM to obtain high-resolution structures of luteovirid particles. *Structure* **27**, 1761-1770.e1763, doi:10.1016/j.str.2019.09.010 (2019).

2 Adams, M. C., Schiltz, C. J., Heck, M. L. & Chappie, J. S. Crystal structure of the potato leafroll virus coat protein and implications for viral assembly. *Journal of Structural Biology* **214**, 107811 (2022).

3 Holm, L. & Rosenstrom, P. DALI server: conservation mapping in 3D. *Nucleic Acids Res* **38**, W545-549, doi:10.1093/nar/gkq366 (2010).

4 Stothard, P. The Sequence Manipulation Suite: JavaScript Programs for Analyzing and Formatting Protein and DNA Sequences. *Biotechniques* **28**, 1102-1104, doi:10.2144/00286ir01 (2000).

5 Boissinot, S., Erdinger, M., Monsion, B., Ziegler-Graff, V. & Brault, V. Both structural and non-structural forms of the readthrough protein of cucurbit aphid-borne yellows virus are essential for efficient systemic infection of plants. *PLoS ONE* **9**, e93448, doi:10.1371/journal.pone.0093448 (2014).

6 Ashkenazy, H. *et al.* ConSurf 2016: an improved methodology to estimate and visualize evolutionary conservation in macromolecules. *Nucleic Acids Res* **44**, W344-350, doi:10.1093/nar/gkw408 (2016).

7 Peter, K. A., Liang, D., Palukaitis, P. & Gray, S. M. Small deletions in the potato leafroll virus readthrough protein affect particle morphology, aphid transmission, virus movement and accumulation. *J Gen Virol* **89**, 2037-2045, doi:10.1099/vir.0.83625-0 (2008).

8 Brault, V. *et al.* Effects of point mutations in the readthrough domain of the beet western yellows virus minor capsid protein on virus accumulation in planta and on transmission by aphids. *Journal of virology* **74**, 1140-1148 (2000).
