# Supplementary material for: Polerovirus N-terminal readthrough domain structures reveal molecular strategies for mitigating virus transmission by aphids
Source: Nat Commun. 2022 Oct 26;13:6368. doi: 10.1038/s41467-022-33979-2 (PMC9606263; doi:10.1038/s41467-022-33979-2)
Supplement: Supplementary file 3 — Reporting Summary [file 41467_2022_33979_MOESM3_ESM.pdf]

nature portfolio

Corresponding author(s): Joshua S. Chappie & Michelle L. Heck

Last updated by author(s): Oct 5, 2022

Reporting Summary

Nature Portfolio wishes to improve the reproducibility of the work that we publish. This form provides structure for consistency and transparency in reporting. For further information on Nature Portfolio policies, see our Editorial Policies and the Editorial Policy Checklist.

Statistics

For all statistical analyses, confirm that the following items are present in the figure legend, table legend, main text, or Methods section.

n/a	Confirmed	
☐	☒	The exact sample size (n) for each experimental group/condition, given as a discrete number and unit of measurement
☐	☒	A statement on whether measurements were taken from distinct samples or whether the same sample was measured repeatedly
☐	☒	The statistical test(s) used AND whether they are one- or two-sided<br>*Only common tests should be described solely by name; describe more complex techniques in the Methods section.*
☒	☐	A description of all covariates tested
☐	☒	A description of any assumptions or corrections, such as tests of normality and adjustment for multiple comparisons
☐	☒	A full description of the statistical parameters including central tendency (e.g. means) or other basic estimates (e.g. regression coefficient) AND variation (e.g. standard deviation) or associated estimates of uncertainty (e.g. confidence intervals)
☐	☒	For null hypothesis testing, the test statistic (e.g. F, t, r) with confidence intervals, effect sizes, degrees of freedom and P value noted<br>*Give P values as exact values whenever suitable.*
☒	☐	For Bayesian analysis, information on the choice of priors and Markov chain Monte Carlo settings
☒	☐	For hierarchical and complex designs, identification of the appropriate level for tests and full reporting of outcomes
☒	☐	Estimates of effect sizes (e.g. Cohen's d, Pearson's r), indicating how they were calculated

Our web collection on statistics for biologists contains articles on many of the points above.

Software and code

Policy information about availability of computer code

Data collection	X-ray data collection was carried out remotely using the NE-CAT RAPD pipeline v2.0.1 for autoindexing and strategy incorporating LABELIT (in PHENIX v1.14), RADDOSE v2, BEST v3.4.4, Mosflm v.7.2.2, and Xtriage (in PHENIX v1.14). Microscopy images were captured using LAS AF version 2.6.0. Digital droplet counts were collected using QuantaSoft v1.7.
Data analysis	XDS (BUILT=20180319) and AIMLESS v0.05.32 (as part of the NE-CAT RAPD pipeline v.2.0.1) were used to process collected X-ray diffraction data.<br>SHELX (C/D v2013/2, E v2016/3) and PHENIX v1.14 were used for SAD phasing.<br>COOT (v0.8.8) and PHENIX v1.14 were used for model building and refinement.<br>PHASER (in PHENIX v1.14 package) was used for molecular replacement.<br>Pymol v1.7 and v.2.1 was used for visual presentation of the models.<br>UCSF Chimera v1.16 was used for structural superposition, molecular modeling, and visualization.<br>Astra VI v.6.1 was used for analysis of SEC-MALS data.<br>QuantaSoft v1.7 (Bio-Rad) was used to analyze digital PCR data.<br>KaleidaGraph v5.01 was used for plotting aphid transmission and mortality data.<br>R version 3.6.3 was used for statistical analysis.

For manuscripts utilizing custom algorithms or software that are central to the research but not yet described in published literature, software must be made available to editors and reviewers. We strongly encourage code deposition in a community repository (e.g. GitHub). See the Nature Portfolio guidelines for submitting code & software for further information.

Data

Policy information about availability of data

All manuscripts must include a data availability statement. This statement should provide the following information, where applicable:

- Accession codes, unique identifiers, or web links for publicly available datasets
- A description of any restrictions on data availability
- For clinical datasets or third party data, please ensure that the statement adheres to our policy

The atomic coordinates of the TuYV and PLRV NRTD structures are deposited in the Protein Data Bank with accession numbers 7ULN [http://doi.org/10.2210/pdb7ULN/pdb] and 7ULO [http://doi.org/10.2210/pdb7ULO/pdb], respectively. The atomic coordinates for the tomato bushy stunt virus coat protein, the turnip yellow virus coat protein, potato leafroll virus coat protein, S. pneumoniae PepA, T. brucei F1-ATPase, Norovirus Saga GII-4 P domain, and A. pernix IF5B that were used for structural comparisons are publicly available from the Protein Data Bank under the accession numbers 2TBV [http:doi.org/10.2210/pdb2TBV/pdb], 6RTK [http://doi.org/10.2210/pdb6RTK/pdb], 6SCO [http://doi.org/10.2210/pdb6SCO/pdb], 3KL9 [http://doi.org/10.2210/pdb3KL9/pdb], 65FD [http://doi.org/10.2210/pdb6F5D/pdb], 6H9V [http://doi.org/10.2210/pdb6H9V/pdb], and 5FG3 [http://doi.org/10.2210/pdb5FG3/pdb], respectively. All amino acid sequences used for alignment and structural conservation mapping were obtained from the publicly accessible Kyoto Encyclopedia of Genes and Genomes (KEGG) database [https://www.genome.jp/kegg/] (see Fig. S5 for individual sequence IDs). Raw data and images generated in this study associated with PLRV transmission efficiency (Fig. 4) and aphid mortality (Fig. 5) are available in the Source Data file or in the Supplementary Information. All reagents are available from the corresponding authors upon reasonable request. Correspondence and requests for materials should be addressed to J.S.C. (chappie@cornell.edu) and M.L.H. (mlc68@cornell.edu).

Field-specific reporting

Please select the one below that is the best fit for your research. If you are not sure, read the appropriate sections before making your selection.

☒ Life sciences ☐ Behavioural & social sciences ☐ Ecological, evolutionary & environmental sciences

For a reference copy of the document with all sections, see nature.com/documents/nr-reporting-summary-flat.pdf

Life sciences study design

All studies must disclose on these points even when the disclosure is negative.

Sample size	The resolution of crystal structure depends on the intrinsic order and X-ray diffraction exhibited by individual protein crystals and the phase information that can be obtained. Hundreds of crystals were screened to optimize diffraction and maximize the anomalous signal at the selenium edge for phasing. The best datasets were used for structure determination and refinement.<br>Samples size for aphid experiments are described in the Methods. No statistical methods were used to predetermine sample size and rather sample size is based on established norms in the field and availability of biological materials (insects, plants, etc.)
Data exclusions	No data were excluded from the analyses.
Replication	Hundreds of protein crystals were similarly screened for X-ray diffraction and anomalous signal and the best datasets are reported and were used for phasing, structure determination, and refinement as described in the Methods.<br>For experiments involving transmission of virus to plants, each individual plant that is inoculated is considered a replicate. For insect mortality experiments, each individual insect is considered a replicate. All aphid experiments were repeated independently at least twice (exact number of replicates for each experiment listed in the Methods). The outcome of each individual aphid experiment is documented in Supplementary Tables S3, S5, and S7.<br>Tests for in planta expression of NRTD were repeated independently at least twice. Western blot analysis and RT-PCR for each experiment were repeated multiple times to achieve publication quality images but the results were the same each time. Microscopic examination of transgenic potato leaves was repeated three times with similar results. Information about sample size and replication is included in the Methods and corresponding figure legends.
Randomization	A random set of diffraction data is set aside during crystallographic refinement for comparison to the model and used to generate the R-free statistic. Selection of these individual reflections is carried out automatically by the software and maintained throughout the refinement procedure.<br>For experiments involving plants and insects, individual plants or insects were randomly assigned to treatments. These insects are asexually reproducing and therefore, clonal. All plants used in an experiment were planted at the same time and grown together in the same growth chamber to minimize variation.
Blinding	Bias introduced during crystallographic model building is accounted for in the established statistical analysis incorporated into refinement procedures. This data is included in Table S1.<br>Data collection is blinded for DAS-ELISA analysis for which 96-well plates are used and wells are only correlated to the relevant samples after the fact. This applies to data collection for all aphid transmission experiments (Fig. 4a,b,d). Data collection was also blinded for digital PCR which also uses a 96-well format (Fig. 4c).

Reporting for specific materials, systems and methods

We require information from authors about some types of materials, experimental systems and methods used in many studies. Here, indicate whether each material, system or method listed is relevant to your study. If you are not sure if a list item applies to your research, read the appropriate section before selecting a response.

Materials & experimental systems

n/a	Involved in the study
☐	☒ Antibodies
☒	☐ Eukaryotic cell lines
☒	☐ Palaeontology and archaeology
☐	☒ Animals and other organisms
☒	☐ Human research participants
☒	☐ Clinical data
☒	☐ Dual use research of concern

Methods

n/a	Involved in the study
☒	☐ ChIP-seq
☒	☐ Flow cytometry
☒	☐ MRI-based neuroimaging

Antibodies

Antibodies used	A commercial Potato leafroll virus antibody (Agdia Cat# SRA30002), a commercial GFP antibody (Abcam Cat# ab6556), a commercial goat anti-rabbit HRP (Promega Cat#W4011), and an anti-NRTD antibody generated in this manuscript.
Validation	Commercial PLRV antibody from Agdia (SRA 30002): "This ELISA test is a qualitative serological assay for the detection of Potato leafroll virus (PLRV) in potato leaf, petiole, sprout, and stem tissue... This assay was designed to detect all strains and isolates of PLRV. Thirty-five distinct samples of PLRV have been experimentally proven to be detected..." from the Full Validation Report for PLRV ELISA kit on the Agdia website.<br>Commercial GFP antibody from Abcam (ab6556): "GFP antibody (ab6556) is reactive against all variants of Aequorea victoria GFP such as S65T-GFP, RS-GFP, YFP, CFP, RFP and EGFP.... Suitable for: IHC-P, Electron Microscopy, ICC, IP, Flow Cyt, IHC-Fr, WB." from the product page on the Abcam website.<br>Commercial goat anti-rabbit HRP from Promega (W4011): "Polyclonal secondary antibody raised in goat, immunoaffinity-purified using immobilized antigens and conjugated to horseradish peroxidase (HRP) enzyme...The Anti-Rabbit IgG (H+L), HRP Conjugate, antibody binds to both heavy and light chains for all rabbit IgG subclasses. As with all antibodies, in certain applications some species-dependent antigen-dependent cross-reactivity may be observed... A starting working dilution of 1:2,500 is suggested for most Western blot, dot blot and ELISA applications." from the product webpage on the Promega website.<br>Custom anti-NRTD antibody: This antibody was generated by Cocalico Biologicals (Stevens, PA, USA) by injecting a rabbit with crystallography-grade purified NRTD protein. The antibody was cross absorbed against E. coli, N. benthamiana, and M. persicae protein homogenates to remove non-specific antibodies. The antibody was purified via sodium sulfate precipitation and specificity was verified by western blot analysis against E. coli, N. benthamiana, and M. persicae protein homogenates, using the purified NRTD and purified PLRV as positive controls.

Animals and other organisms

Policy information about studies involving animals; ARRIVE guidelines recommended for reporting animal research

Laboratory animals	Lab-reared colonies of the green peach aphid, Myzus persicae, were used in this study. These are wild-type individuals originally collected from New York state and maintained on Physalis floridana plants in the growth chamber at 20C with 12-hour light/12-hour dark photoperiod. The colony consists of pathogenetically-reproducing females. Fourth instar and adult individuals were used for experiments.
Wild animals	The study did not involve wild animals.
Field-collected samples	This study did not involve samples collected from the field.
Ethics oversight	No ethical approval was required since the study animals are invertebrate insects. Use of recombinant DNA molecules and transgenic potato plants was conducted in line with institutional and regulatory approval.

Note that full information on the approval of the study protocol must also be provided in the manuscript.
