# Supplementary material for: Polerovirus N-terminal readthrough domain structures reveal molecular strategies for mitigating virus transmission by aphids
Source: Nat Commun. 2022 Oct 26;13:6368. doi: 10.1038/s41467-022-33979-2 (PMC9606263; doi:10.1038/s41467-022-33979-2)
Supplement: Supplementary file 4 — Source Data file [file 41467_2022_33979_MOESM4_ESM.zip › Schiltz_et_al_raw_images.pdf]

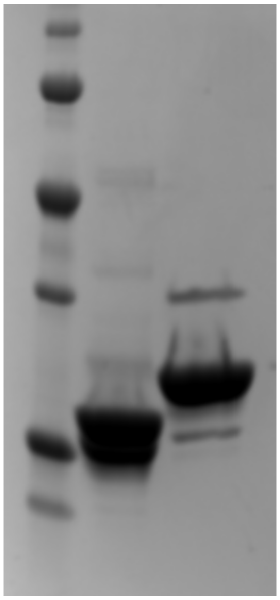

Uncropped SDS-PAGE gel image associated with Fig. S1c showing purified PLRV (middle lane) and TuYV (right lane) ^{N}RTDs with molecular weight markers (left lane; top to bottom: 100 kDa, 75 kDa, 50 kDa, 37 kDa, 25 kDa, 20 kDa).

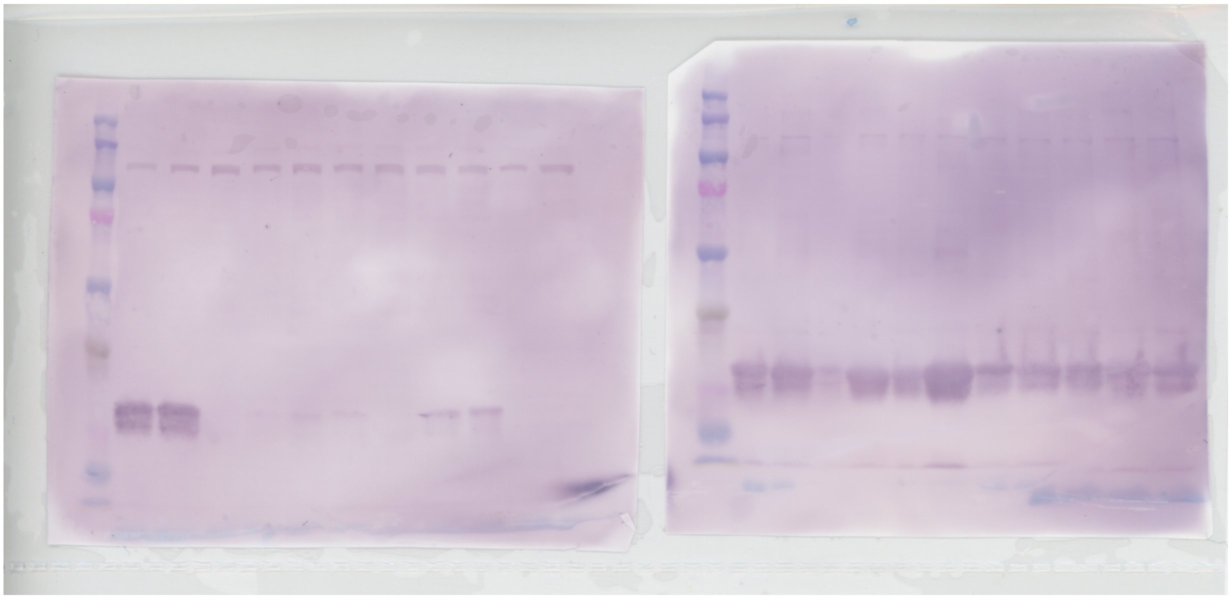

Uncropped gel images associated with Fig. S10d (left) and S10e (right). Western blots performed with anti-^{N}RTD antibody as described in the Methods.

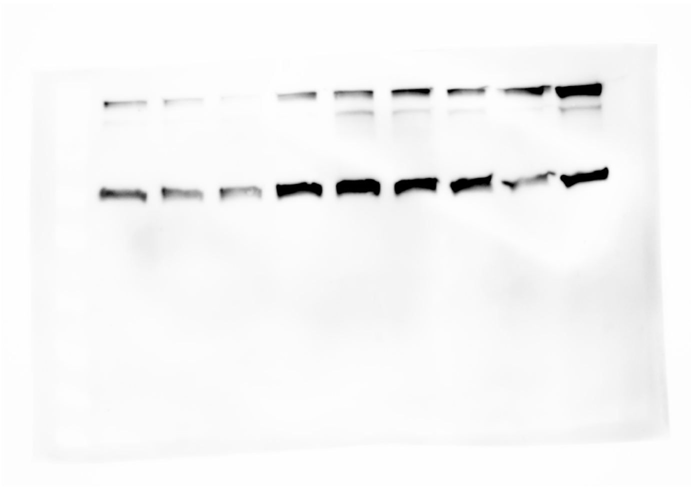

Uncropped gel image associated with Fig. S12b. Western blot performed with anti-^{N}RTD antibody as described in the Methods.

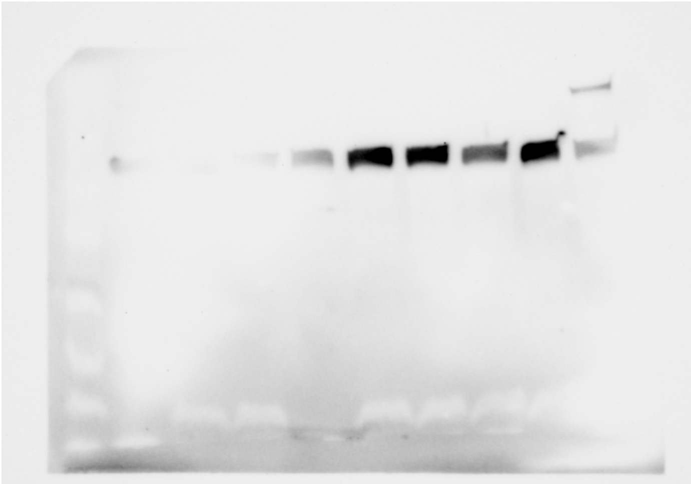

Uncropped gel image associated with Fig. S11c. Western blot performed with anti-^{N}RTD antibody as described in the Methods.

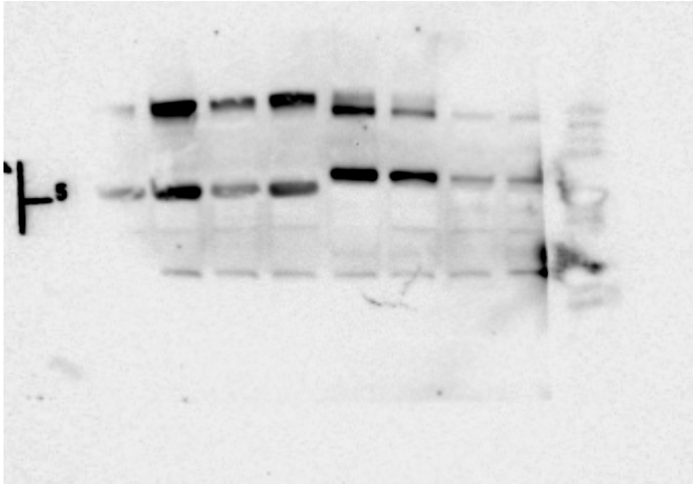

Uncropped gel image associated with Fig. S12d. Western blot performed with anti-GFP antibody as described in the Methods.

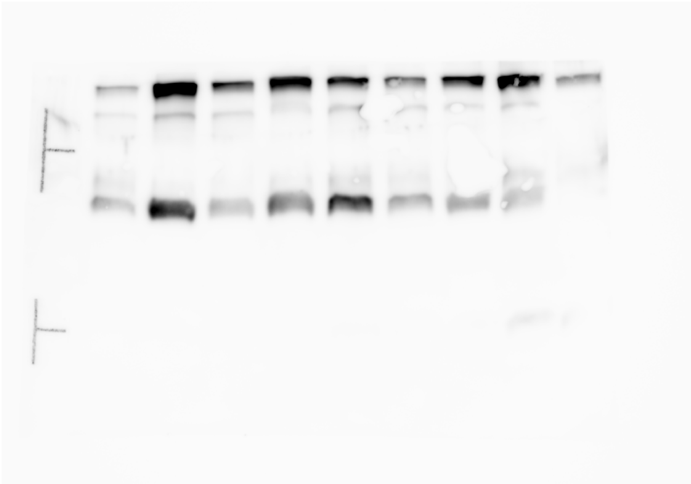

Uncropped gel image associated with Fig. S13a. Western blot performed with anti-^{N}RTD antibody as described in the Methods.

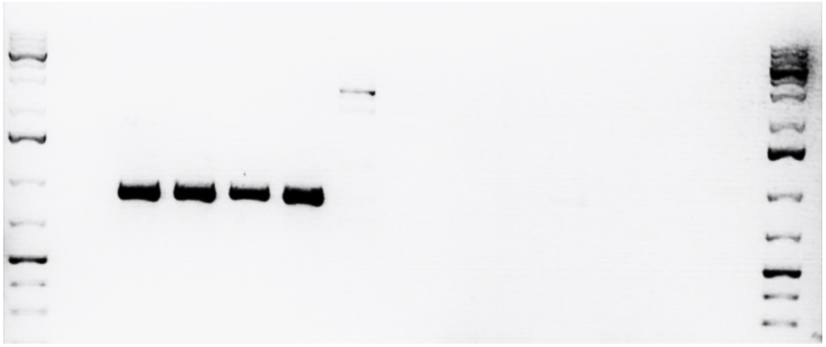

Uncropped gel image associated with Fig. S13b. RT-PCR analysis of transgenic potatoes and controls performed as described in the methods.
